# Supplementary material for: Tuning the role of charge-transfer states in intramolecular singlet exciton fission through side-group engineering
Source: Nat Commun. 2016 Dec 7;7:13622. doi: 10.1038/ncomms13622 (PMC5150654; doi:10.1038/ncomms13622)
Supplement: Supplementary Information — Supplementary Figures 1-27, Supplementary Tables 1-9, Supplementary Notes 1-2 and Supplementary References [file ncomms13622-s1.pdf]

Supplementary Figures

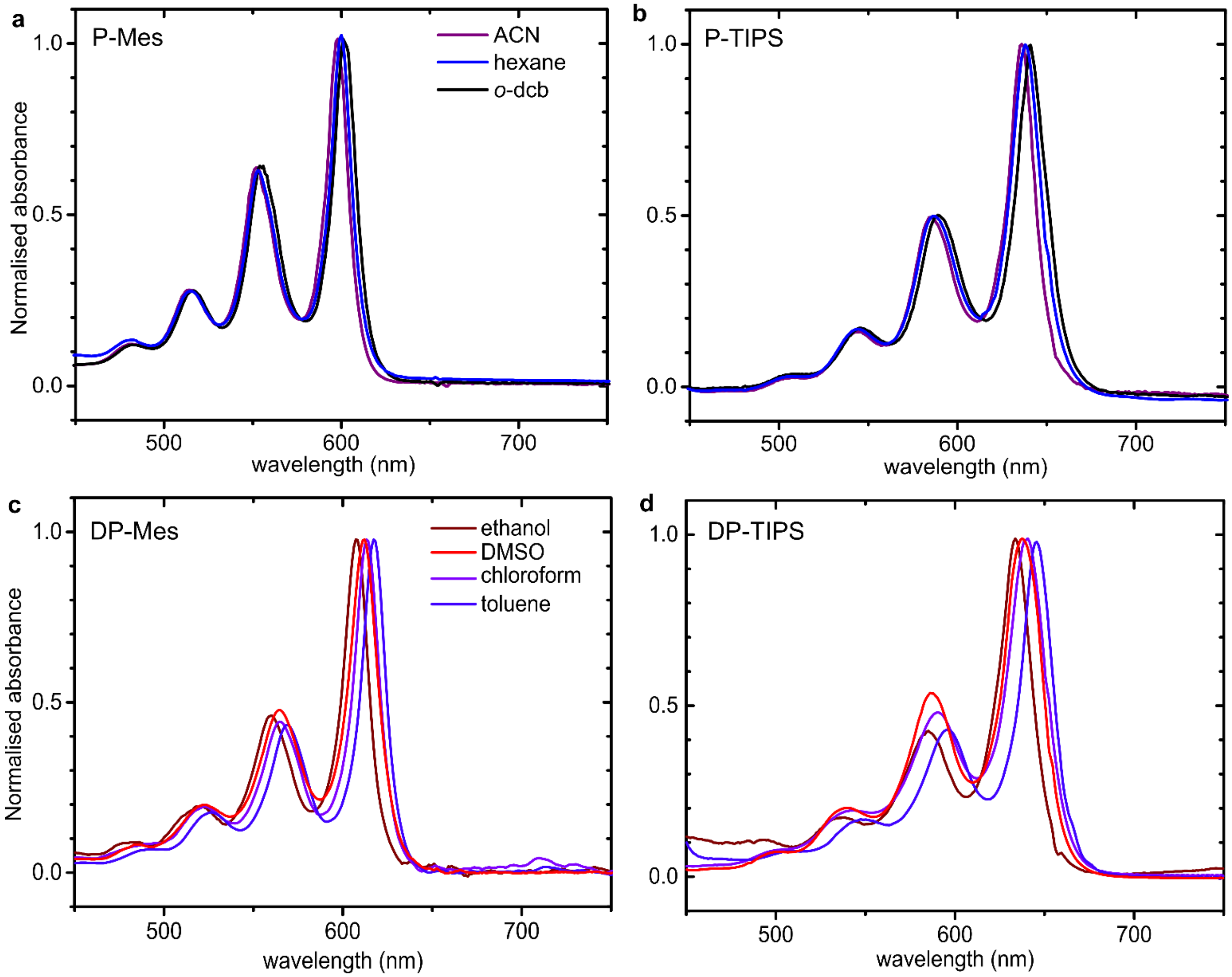

Supplementary Figure 1. Normalised solvent-dependent absorption spectra. (**a**) P-Mes, (**b**) P-TIPS, (**c**) DP-Mes and (**d**) DP-TIPS in solvents of different polarity/polarisability. Absorption spectra of P-Mes and P-TIPS showed negligible change in different solvents. Legends are the same for plots on left and right.

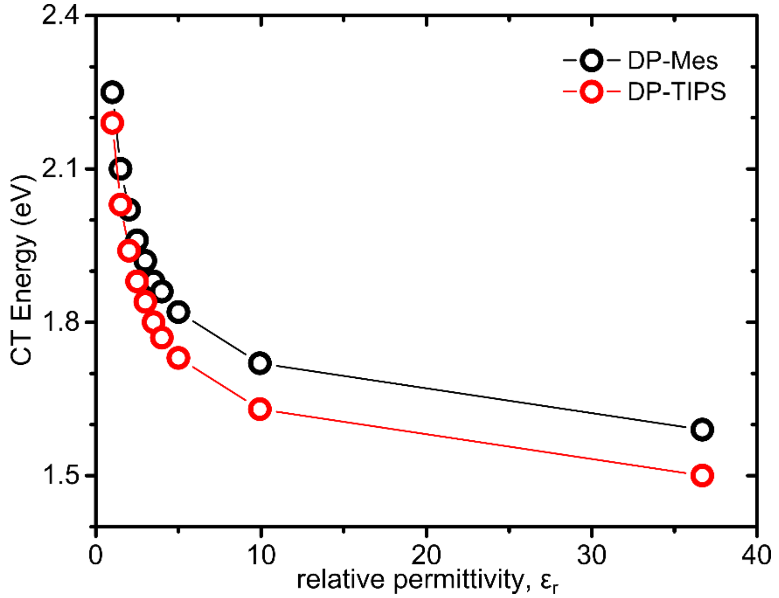

Supplementary Figure 2. Constrained DFT (c-DFT) predicted CT energy of DP-Mes and DP-TIPS as function of medium permittivity.

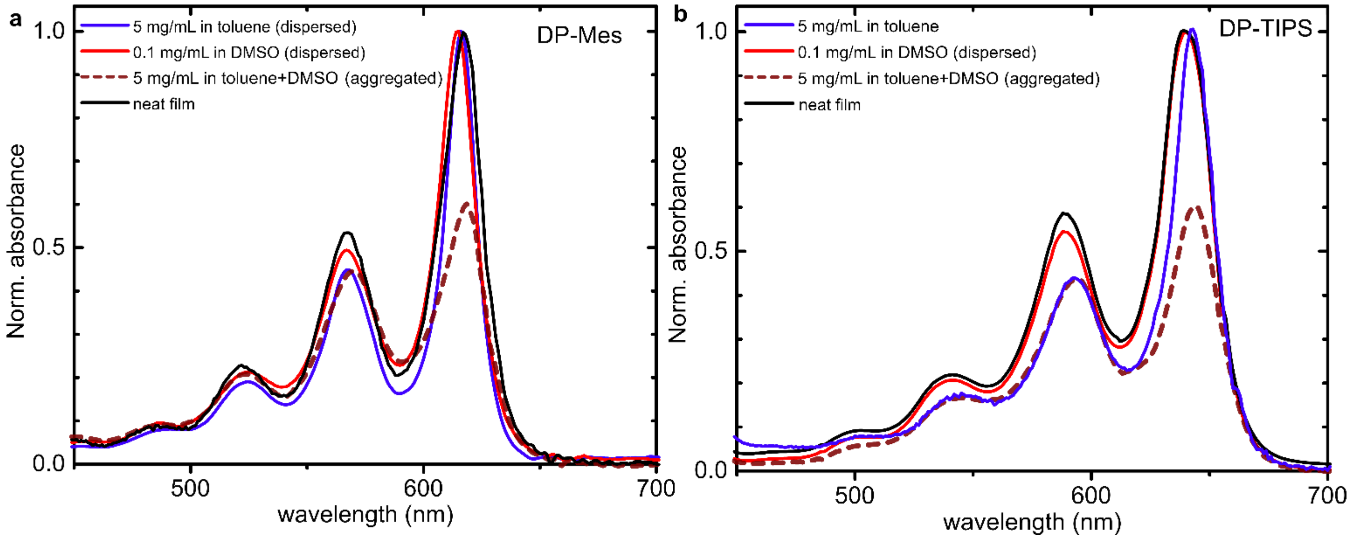

Supplementary Figure 3. Pentacene dimer absorption in condensed phase. Normalised absorption spectra (**a**) DP-Mes and (**b**) DP-TIPS in non-polar solvent toluene (blue), polar solvent DMSO (red), aggregated molecules (wine), and neat film (black). Aggregation is induced by adding polar solvent into highly concentrated solution, which is fully dissolved in non-polar solvents. The aggregated spectra is rescaled to the absorption peak at ~580 nm to highlight the reduction in S_0-S_1 transition as aggregation occurs.

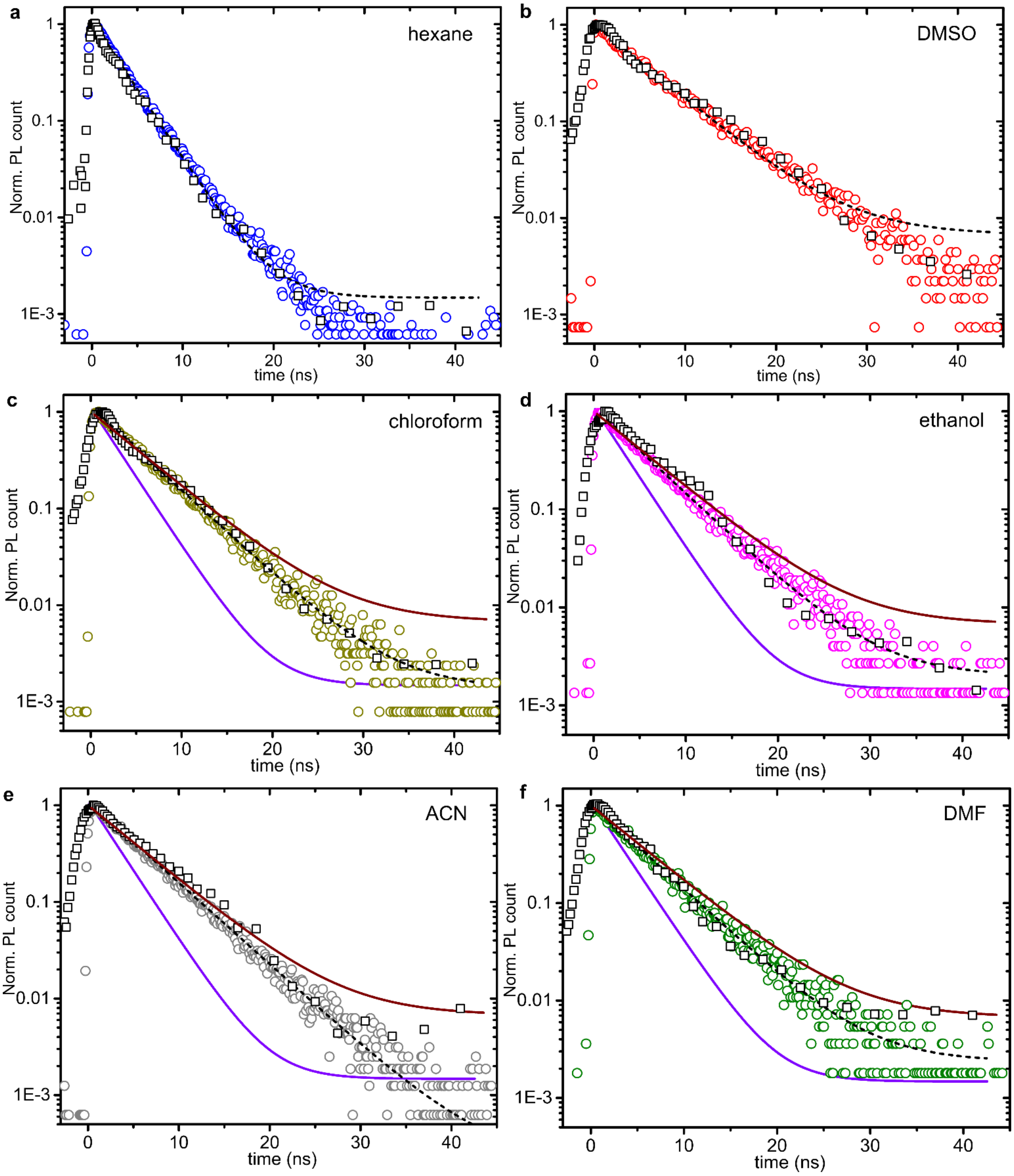

Supplementary Figure 4. Transient PL (650nm) kinetics of DP-Mes in different solvents excited at 470nm. (**a**) PL kinetic of regenerated S* in hexane (**b**) PL kinetic of S_{stab} in DMSO. The solid lines in (**c-f**) are exponential fits to the kinetics of regenerated S* (purple) in hexane and S_{stab} (wine) in DMSO. Dotted lines are single exponential fits to the kinetic in each solvent. Scatter plots (open squares) are kinetics of the corresponding excited state measured in TA. Note the strong agreement on early timescales. The deviation beyond ~30 ns is primarily due to the lower dynamic range in TA than in the PL measurement.

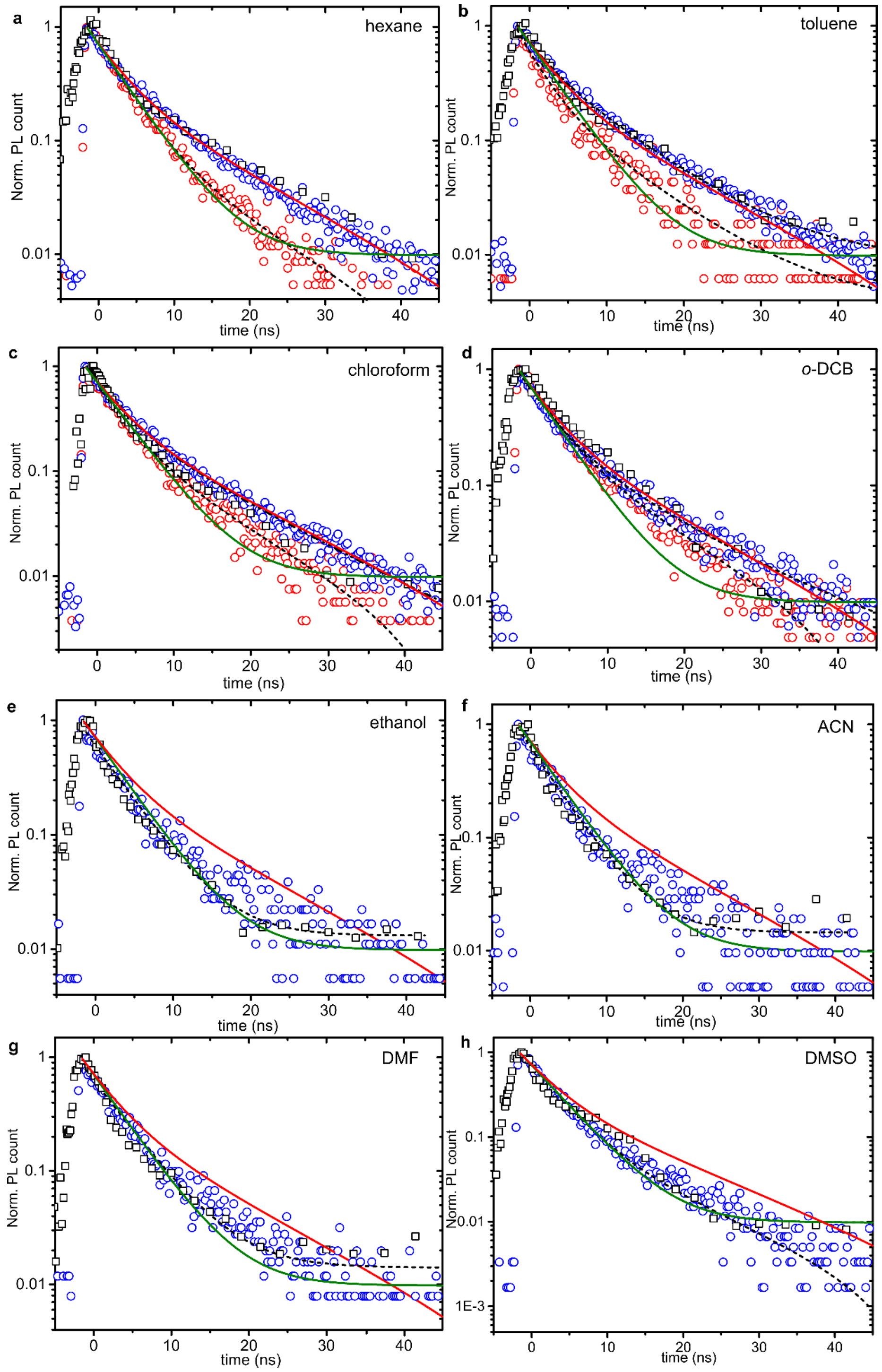

Supplementary Figure 5. Transient PL kinetics of DP-TIPS in different solvents following 470nm excitation. In (**a**) hexane (**b**) toluene (**c**) chloroform and (**d**) *o*-DCB the kinetics are taken at 660 nm (blue dot) and 720 nm (red dot), while in the remaining solvents the kinetics are uniform across the PL spectrum and taken at 660 nm. Solid lines in are exponential fits to the kinetics of S_{stab} (red) in hexane and CT_{stab} (green) in DMSO, the two extremes of kinetic behaviour. Dotted lines are single exponential fitting to the kinetic. Scatter plots (open squares) are kinetics of the long-lived state measured in TA. Note the strong agreement on early timescales. The deviation beyond ~30 ns is primarily due to the lower dynamic range in TA than in the PL measurement.

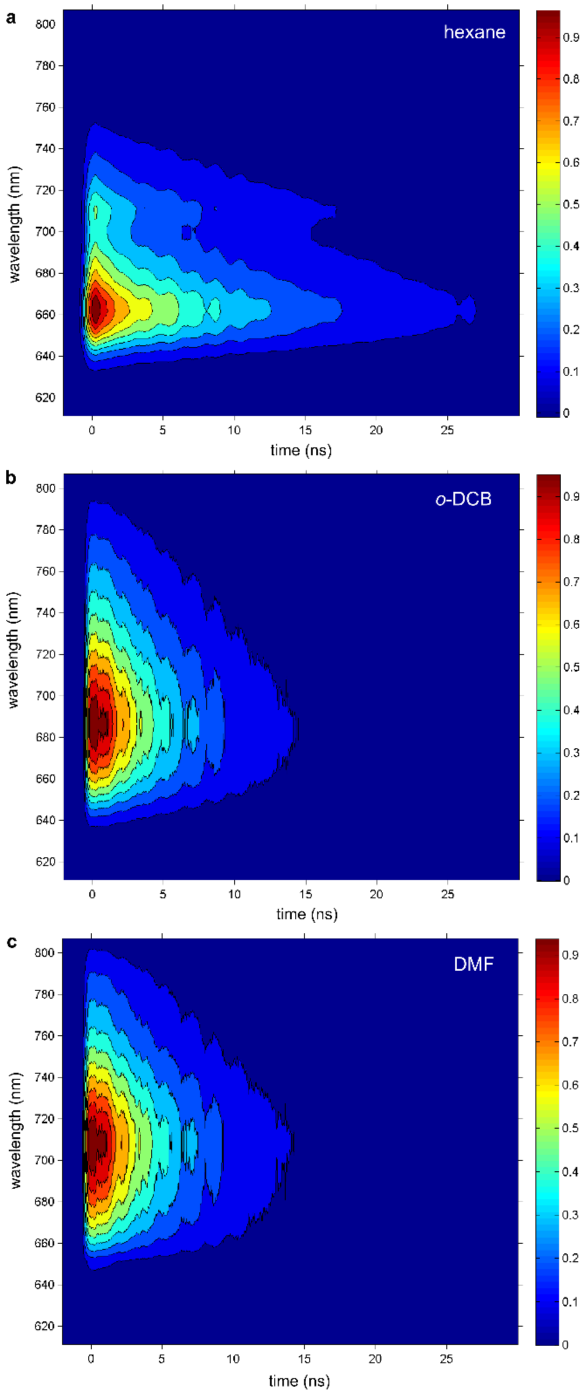

Supplementary Figure 6. Long-time emission in DP-TIPS. Time-correlated single-photon counting decay maps of DP-TIPS in (**a**) hexane, (**b**) *o*-DCB, and (**c**) DMF. Individual PL wavelengths were selected with a monochromator, and kinetics we collected by integration for the same period of time at each wavelength, enabling the construction of PL decay maps on the 1-30 ns timescale. The species detected in this regime have failed to undergo SF, and can be identified by comparison with fast PL spectra and TA kinetics as S_{stab} (hexane) or residual CT (*o*-DCB and DMF).

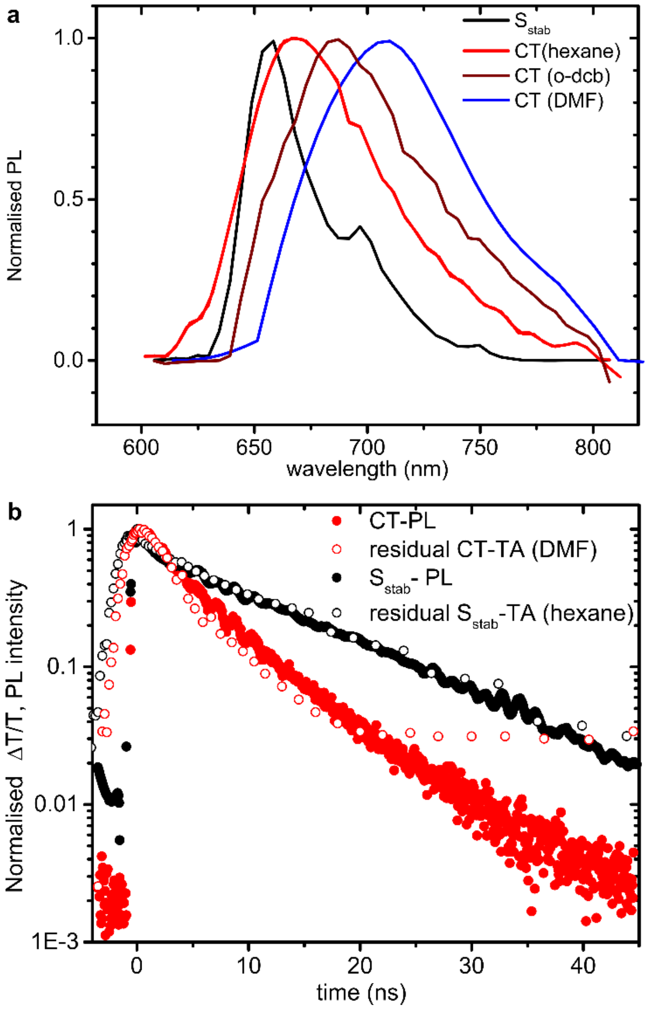

Supplementary Figure 7. Spectral decomposition of PL decay maps. (**a**) Spectra and (**b**) corresponding kinetics of spectrally decomposed of DP-TIPS transient PL data from above, in hexane, *o*-DCB, and DMF. Open circles in (b) are the population kinetics of the associated species collected in TA experiments, confirming the spectral assignment.

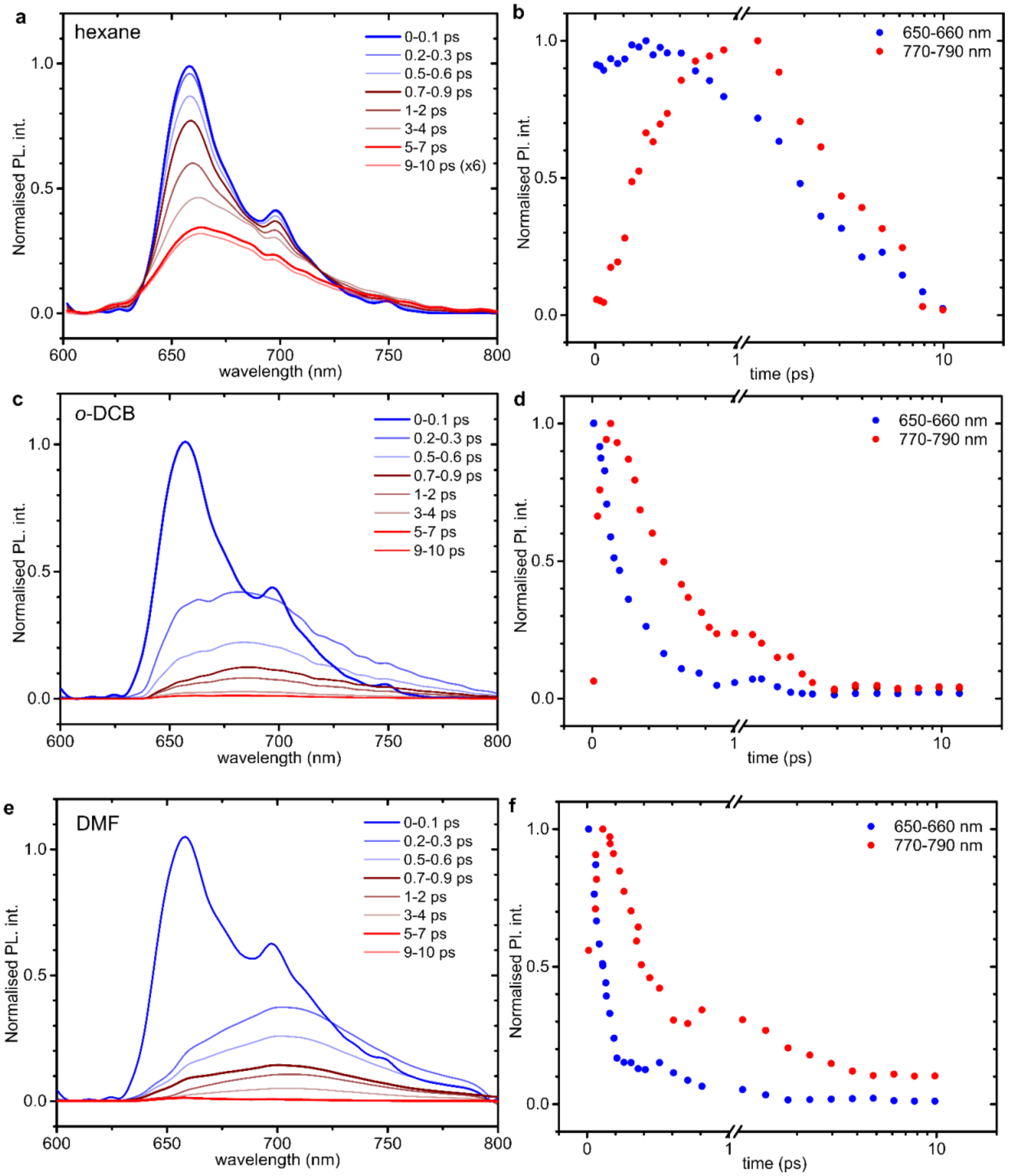

Supplementary Figure 8. CT formation in DP-TIPS. Transient grating PL spectra of DP -TIPS in (**a**) hexane, (**c**) *o*-DCB, and (**e**) DMF and (**b,d,f**) selected kinetics integrated over the indicated spectral regions. The formation of the structureless, red-shifted emissive CT species can be directly resolved in the data. Its spectral position progressively red-shifts with solvent polarity. Raw data sets from TGPL were analysed with Singular Value Decomposition to determine the number of real components and to remove experimental noise. From the relative magnitude of the eigenvalues, we determined that only two significant spectral components were present in the data. Thus only the first two components with the highest eigenvalues are used and plotted here.

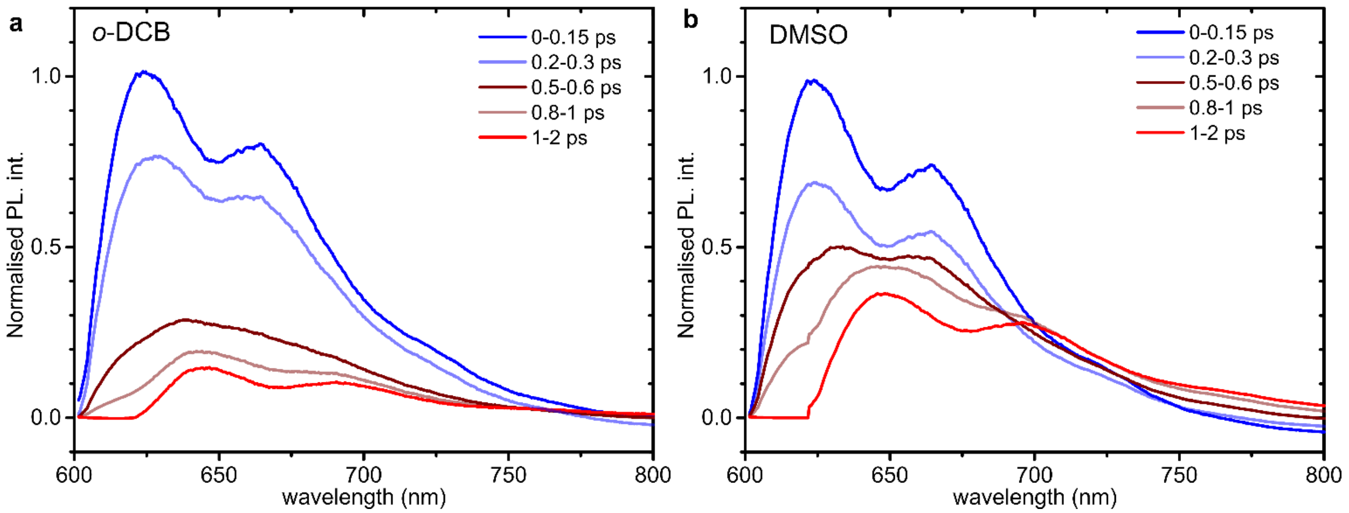

Supplementary Figure 9. **S_{stab} formation in DP-Mes**. Transient grating PL spectra of DP-Mes showing early-time spectral evolution in (**a**) *o*-DCB and (**b**) DMSO. The emission progressively red-shifts as S_{stab} is formed, leading to the long-lived population detected with slower PL techniques.

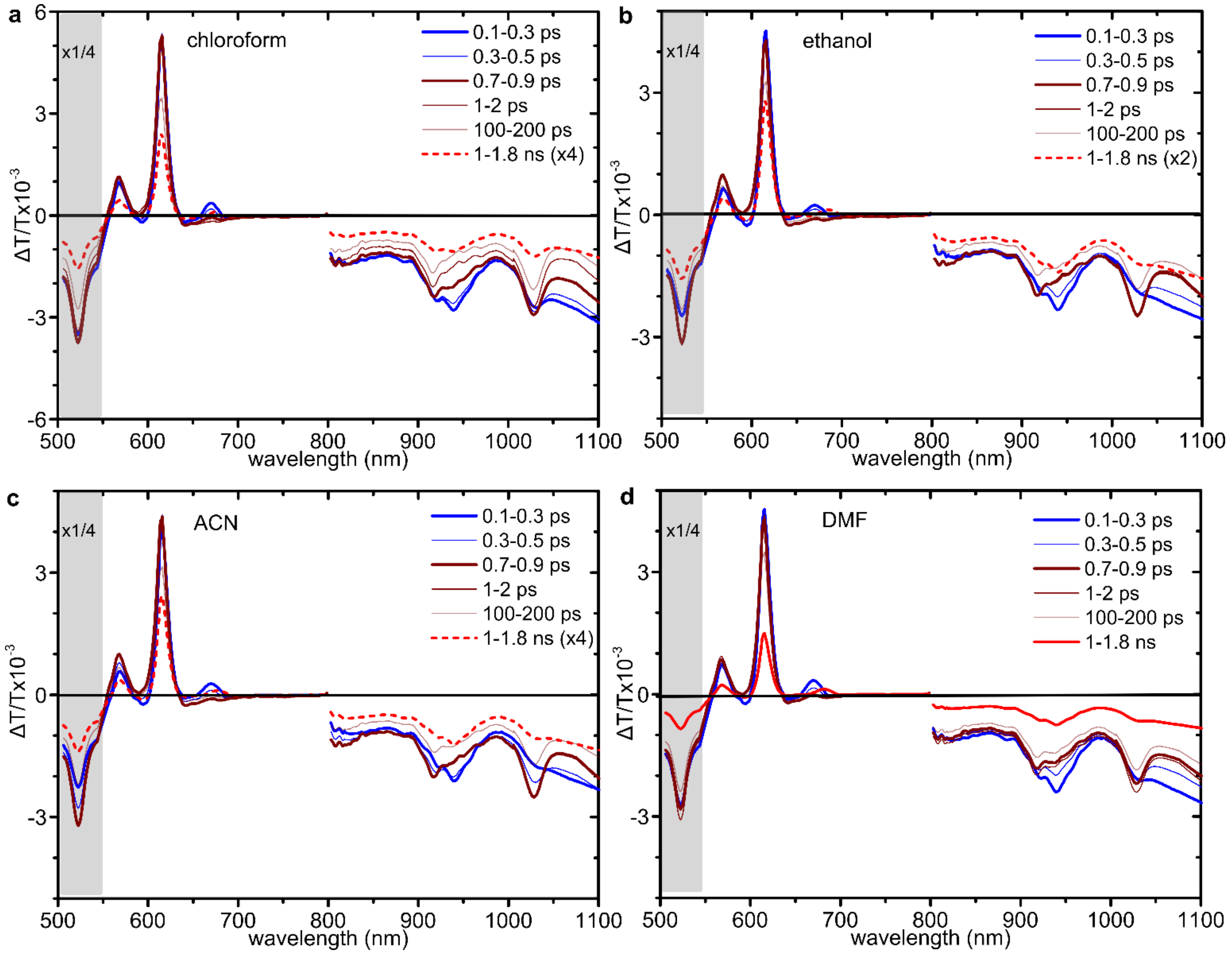

Supplementary Figure 10. Solvent-dependent SF in DP-Mes. TA spectra of DP-Mes in (**a**) chloroform, (**b**) ethanol, (**c**) ACN, and (**d**) DMF at indicated time delays. Dashed lines show spectral slices that have been magnified for clarity. The strong triplet PIA at 500-550 nm has also been scaled by ¼ for clarity.

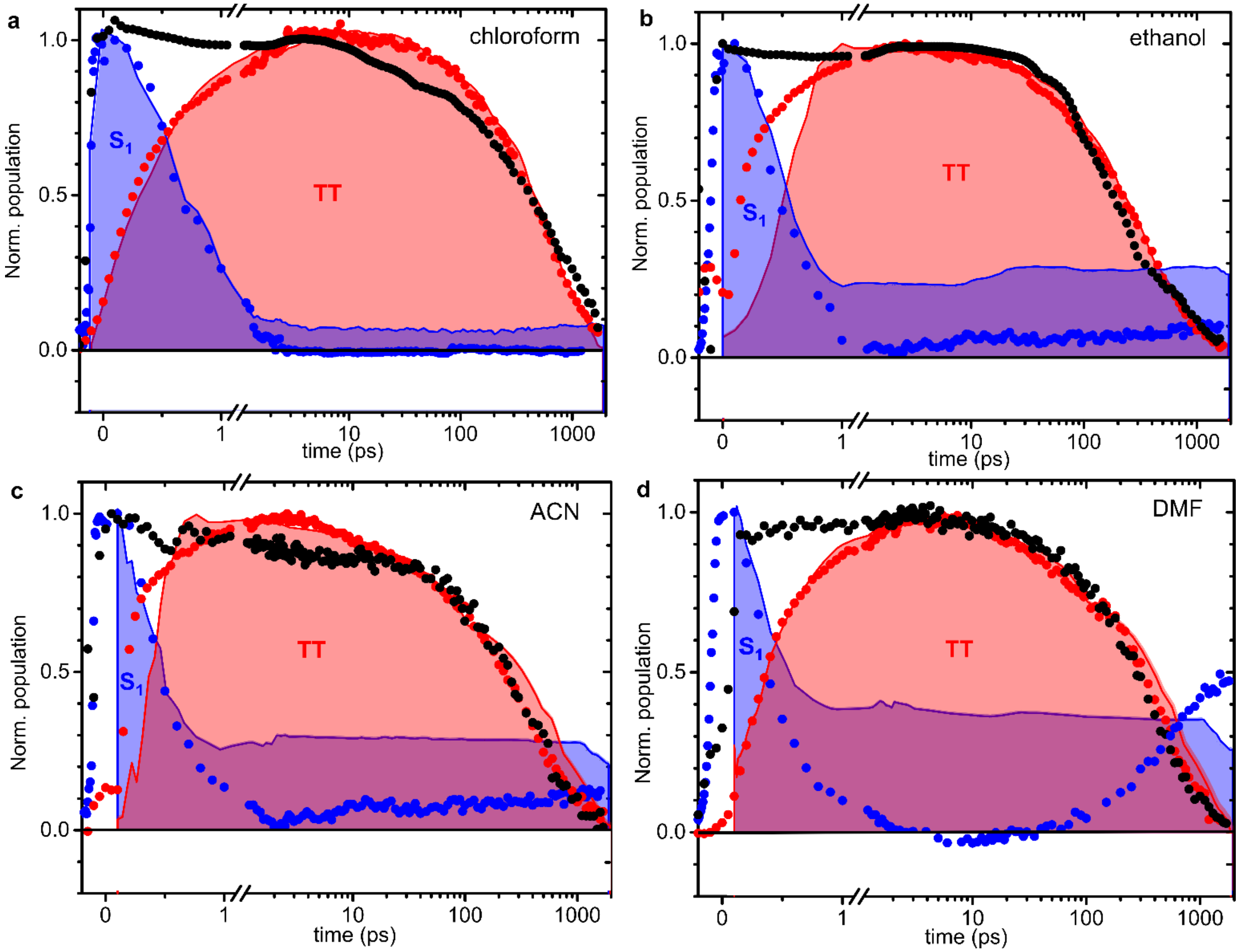

Supplementary Figure 11. Two-species SF dynamics in DP-Mes. Normalised population kinetics of different excited species in DP-Mes in various solvents from spectral decomposition (shaded regions). The scatter plots represent the raw kinetics averaged in the region of GSB (610-620 nm, black), primarily TT PIA (520-530 nm, red) and S_1 SE (665-670nm, blue). We note that the nearly invariant GSB kinetic over the triplet formation timescale indicates that essentially no population is lost during SF: molecules either form triplet pairs or remain in S_{stab}.

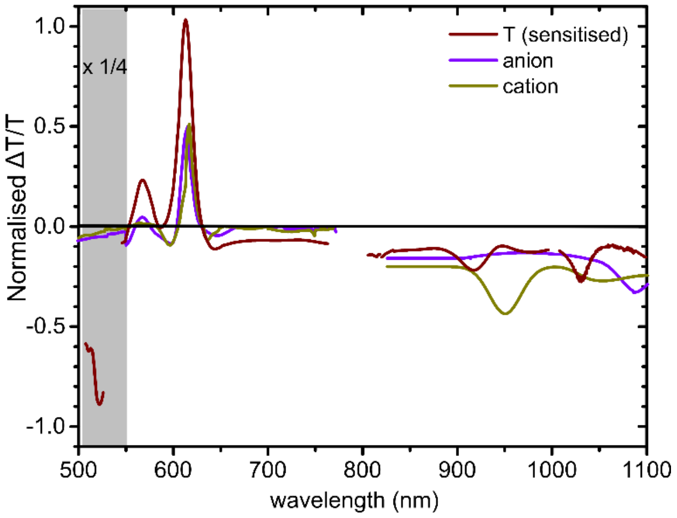

Supplementary Figure 12. DP-Mes reference spectra. Transient absorption features of DP-Mes triplet state, obtained by the same sensitisation protocol as above for DP-TIPS, as well as anion and cation spectra. The latter were obtained by chemical oxidation or reduction of DP-Mes in solution, and were converted into ΔT/T units for comparison.

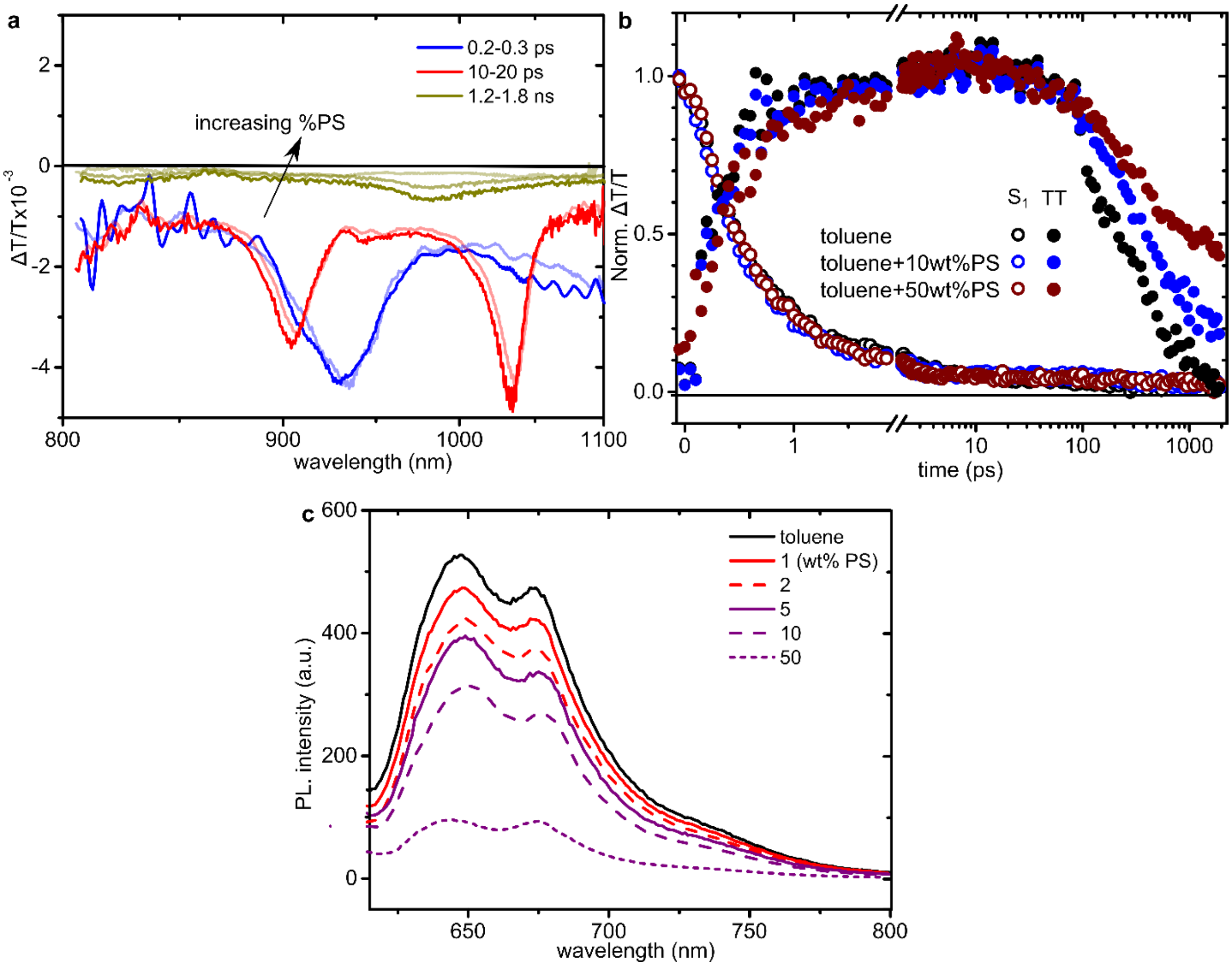

Supplementary Figure 13. Suppressing S* formation through viscosity. (**a**) TA data of DP-Mes at indicated pump-probe delays for different weight ratios of PS in toluene (0%:dark to 50%:light). S* formation (PIA at 975 nm) is inhibited with increasing viscosity. (**b**) The SE kinetic of S_1 (open symbols) in the mixed system shows no change in SF rate and SF efficiency as viscosity increases, but slight increases in T lifetime (filled symbols) are observed. (**c**) The corresponding time-integrated PL spectra as a function of viscosity, showing a strong reduction in intensity due to prevention of S* formation as the viscosity increases.

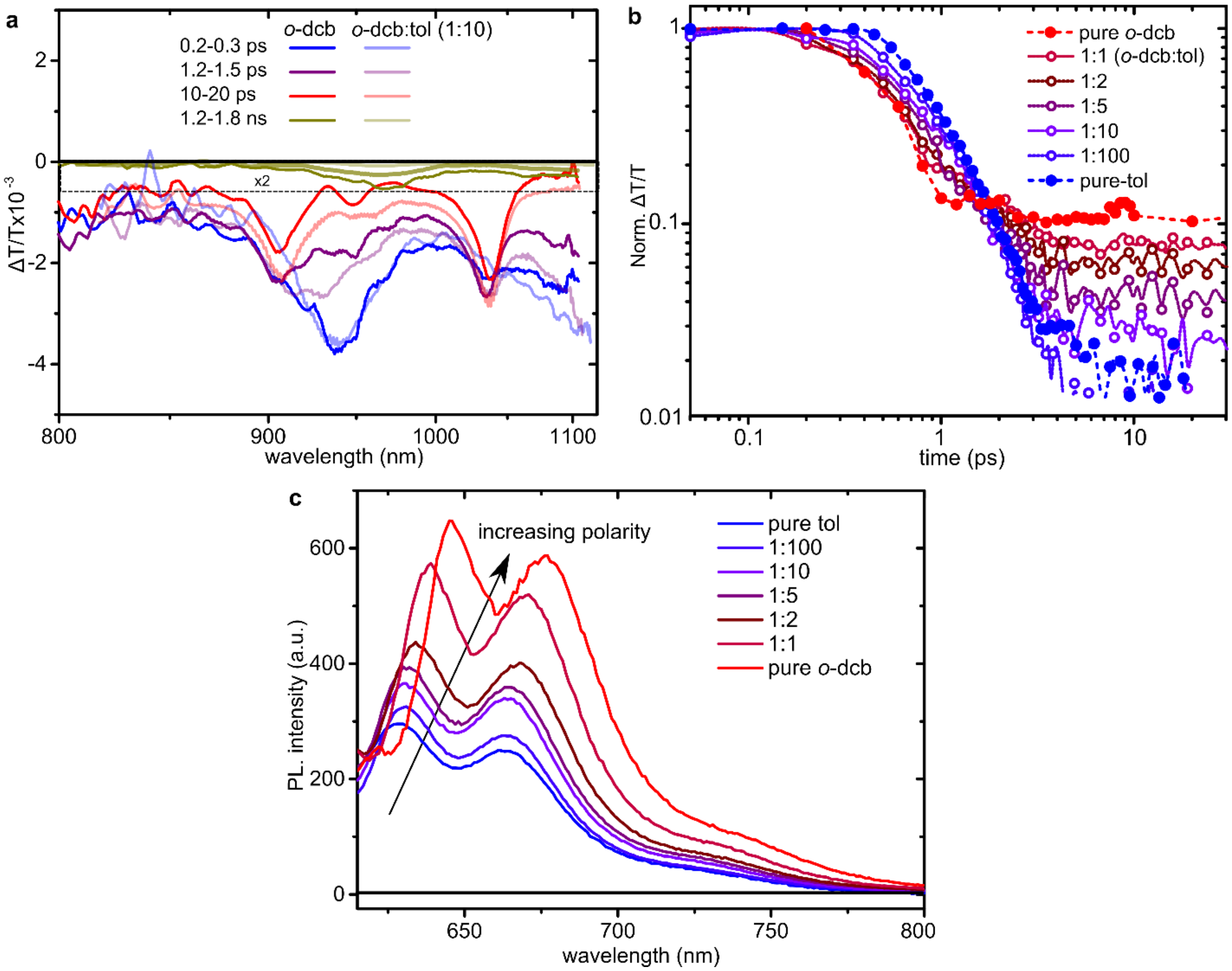

Supplementary Figure 14. Fine-tuning polarity in DP-Mes with co-solvents. (**a**) TA data of DP-Mes at indicated pump-probe delays at different ratios of toluene to *o*-DCB. S* formation is inhibited with increasing polarity. Spectra on the longest timescale are magnified by 2x for clarity. (**b**) The SE kinetic of S_1 in the mixed solvents showing an increase in SF rate and reduction in SF efficiency (increased magnitude of S_{stab} 'tail' beyond 5 ps) as polarity increases. (**c**) The corresponding time-integrated PL spectra as a function of polarity, showing a gradual shift from S* to S_{stab} as polarity increases.

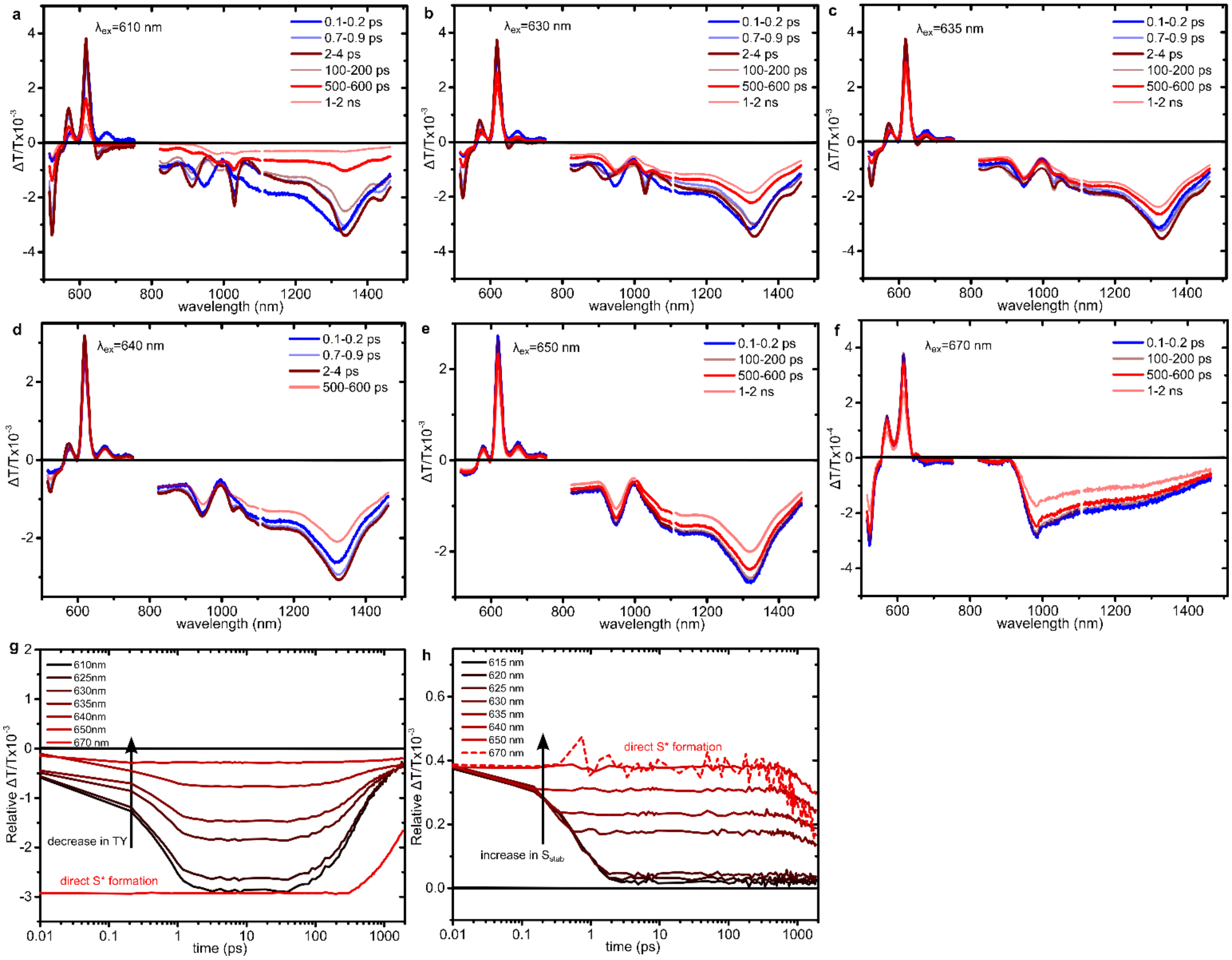

Supplementary Figure 15. Excitation-dependent photophysics of DP-Mes. TA spectra of DP-Mes in hexane following sub-bandgap excitation at (**a**) 610 nm, (**b**) 630 nm, (**c**) 635 nm, (**d**) 640 nm, (**e**) 650 nm, (**f**) 670 nm at the indicated time delays, (**g**) relative kinetic at 510-520 nm (TT PIA), (**h**) 640-650 nm (SE) showing gradual decrease in triplet yield and increase in S_{stab} (up to 650 nm). At the lowest excitation energy (670 nm), a small population of S* can be directly populated. All of the kinetics (except following excitation at 670 nm) are multiplied by a prefactor to make the GSB at each measurement comparable to the GSB following excitation at 610 nm. Kinetics for excitation at 670 nm (x10) have been scaled for clarity.

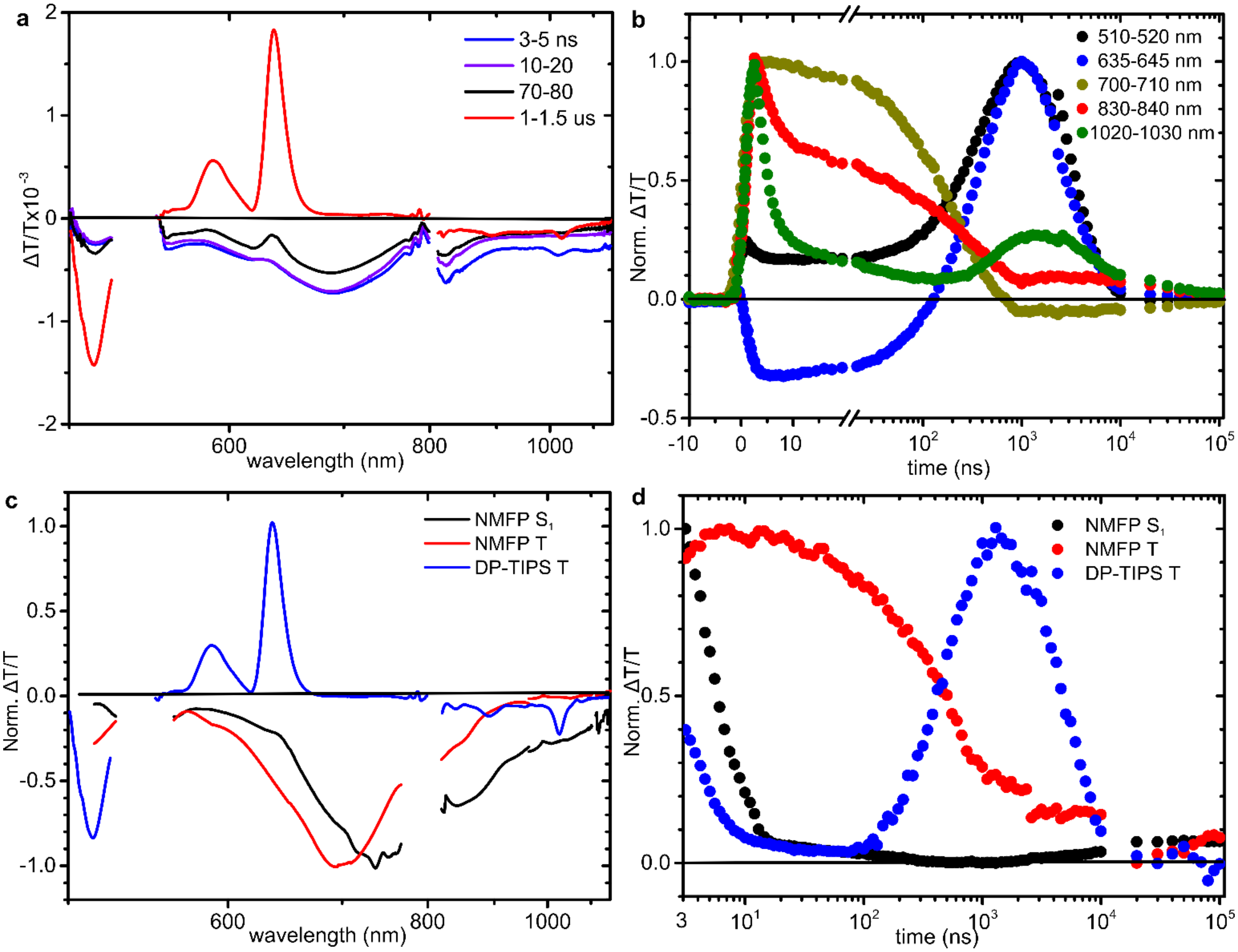

Supplementary Figure 16. Triplet sensitisation of DP-TIPS. In the sensitisation experiment, DP-Mes/DP-TIPS was mixed with the triplet sensitiser N-methylfulleropyrrolidine (NMFP) in toluene. (**a**) Raw TA timeslices of the DP-TIPS/NMFP mixed solution, at the indicated probe delays following nanosecond excitation at 532 nm. There is substantial initial NMFP excitation due to the large excess, and the fullerene exhibits efficient intersystem crossing to generate a large population of triplets, NMFP T, shortly after excitation. This state is characterised by a broad PIA peaked between 700 and 750 nm. Subsequent triplet energy transfer from NMFP T, demonstrated by the growth of pronounced DP-TIPS GSB (positive features) on long timescales, yields long-lived triplet on DP-Mes/DP-TIPS. Any species formed by direct excitation of DP-Mes/DP-TIPS decay with 10's ns time constant or faster, so the triplet signatures observed here on µs timescales cannot arise from direct excitation. (**b**) Kinetics of DP-TIPS sensitisation, integrated over the indicated bands. (**c**) Spectral decomposition of sensitisation spectra, following the same procedures used for sub-ps TA, allows clear determination of the individual spectral components. (**d**) Normalised population kinetics extracted from the spectral decomposition, showing gradual energy transfer from NMFP to DP-TIPS. The signatures of DP-TIPS triplet on very early timescales are due to convolution with the instrument response and can be neglected. An exponential fit to the final triplet PIA decay or to the extracted T population kinetics gives a decay lifetime of 5 µs, the intrinsic lifetime of single triplet excitons on the pentacene dimer, and the ~4 orders of magnitude faster decay we observe through direct excitation must arise through geminate TTA, which in turn confirms the presence of SEF.

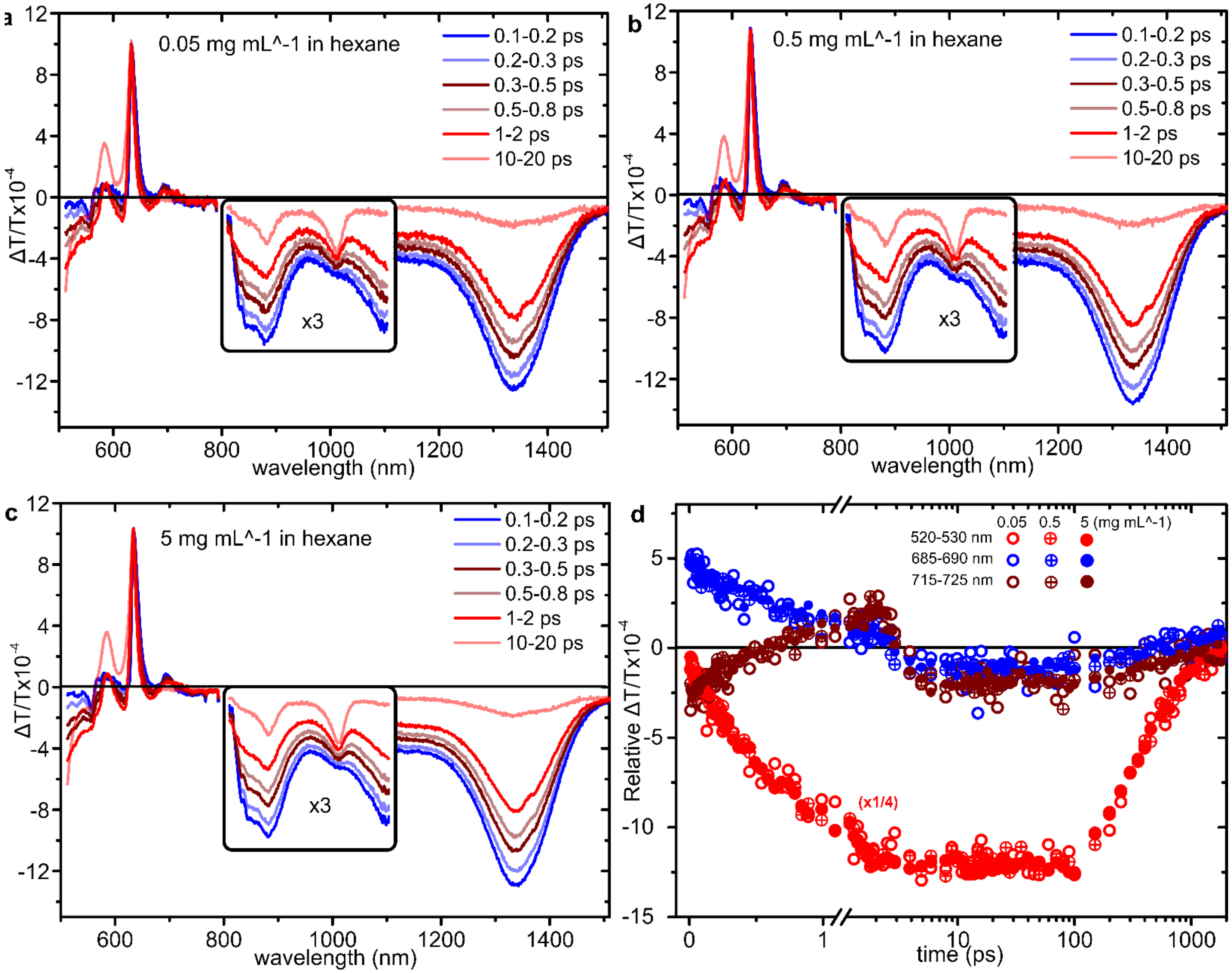

Supplementary Figure 17. Concentration-independent SF in DP-TIPS in non-polar solvent. TA spectra of DP-TIPS in hexane (**a**) 0.05 mg mL^{-1}, (**b**) 0.5 mg mL^{-1}, (**c**) 5 mg mL^{-1} at indicated time delays, (**d**) and the corresponding kinetic at different concentration. The indicated NIR spectral region has been magnified for clarity. The conversion of S_1 into CT, CT into TT and subsequent geminate TTA were independent of concentration.

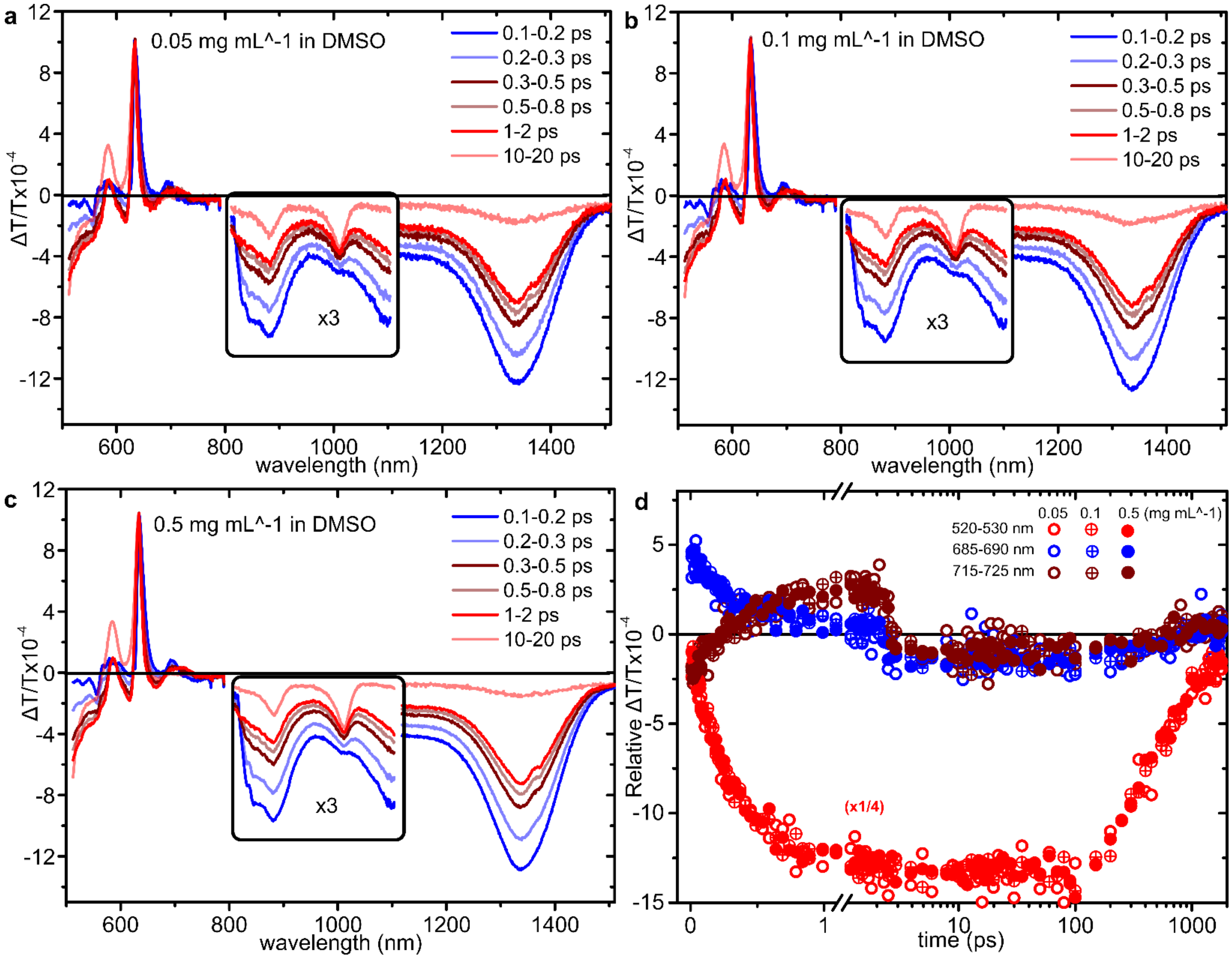

Supplementary Figure 18. Concentration-independent SF in DP-TIPS in polar solvent. TA spectra of DP-TIPS in DMSO (**a**) 0.05 mg mL^{-1}, (**b**) 0.1 mg mL^{-1}, (**c**) 0.5 mg mL^{-1} at indicated time delays, (**d**) and the corresponding kinetic at different concentration. The indicated NIR spectral region has been magnified for clarity. The conversion of S_1 into CT, CT into TT and subsequent geminate TTA were independent of concentration.

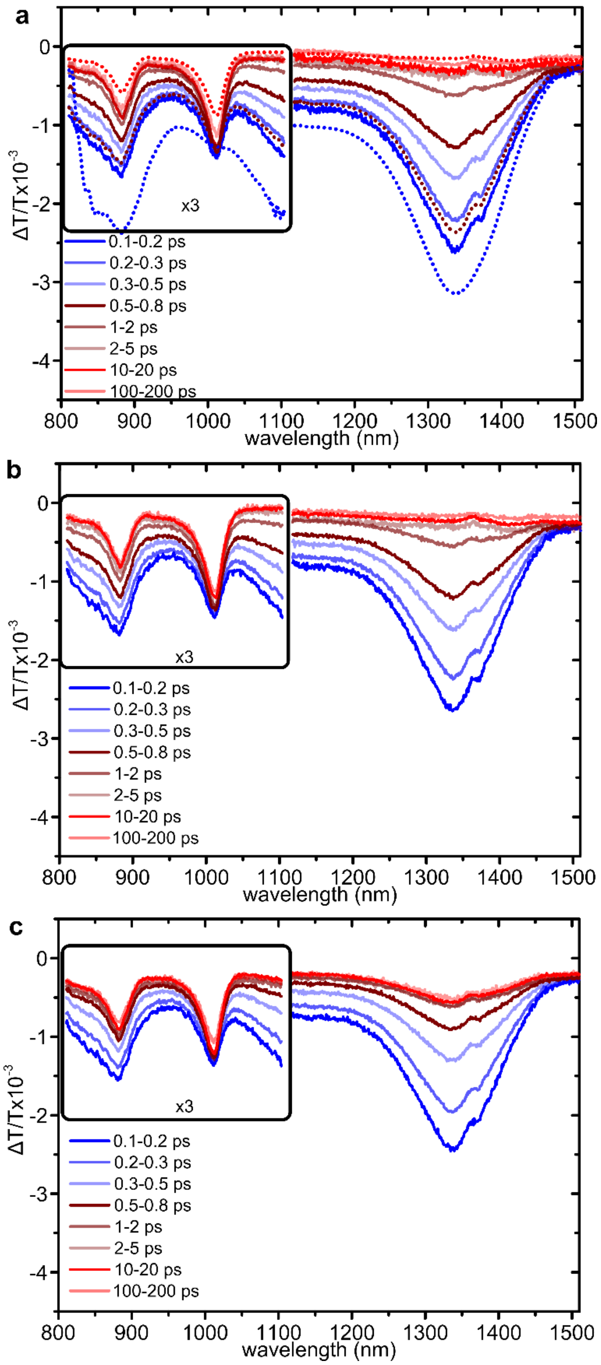

Supplementary Figure 19. Direct excitation of CT. The observation of PL and SE assigned to the CT state show that the radiative decay to the ground state is weakly allowed, suggesting direct excitation of CT should also be possible. TA measurements were performed on DP-TIPS in hexane, *o*-DCB and DMF using sub-gap excitation at 660 nm, 680 nm, and 710 nm. TA spectra of DP-TIPS following sub-gap excitation in (**a**) hexane at 660 nm, (**b**) *o*-DCB at 680 nm and (**c**) DMF at 710 nm. The initial spectral feature in all cases matches the signature of CT rather than S_1. This state proceeds to undergo SF with the same rate as following S_1 excitation. CT could not be directly excited following excitation at 710 nm in either hexane or *o*-DCB. Only in highly polar DMF is the CT state energy low enough for excitation at this wavelength. Sub-gap excitation behaviour is examined in further detail in Supplementary Fig. 22 below. Dashed lines are S_1 (blue), CT (brown) and TT (red) PIA signatures from resonant-excitation TA for reference.

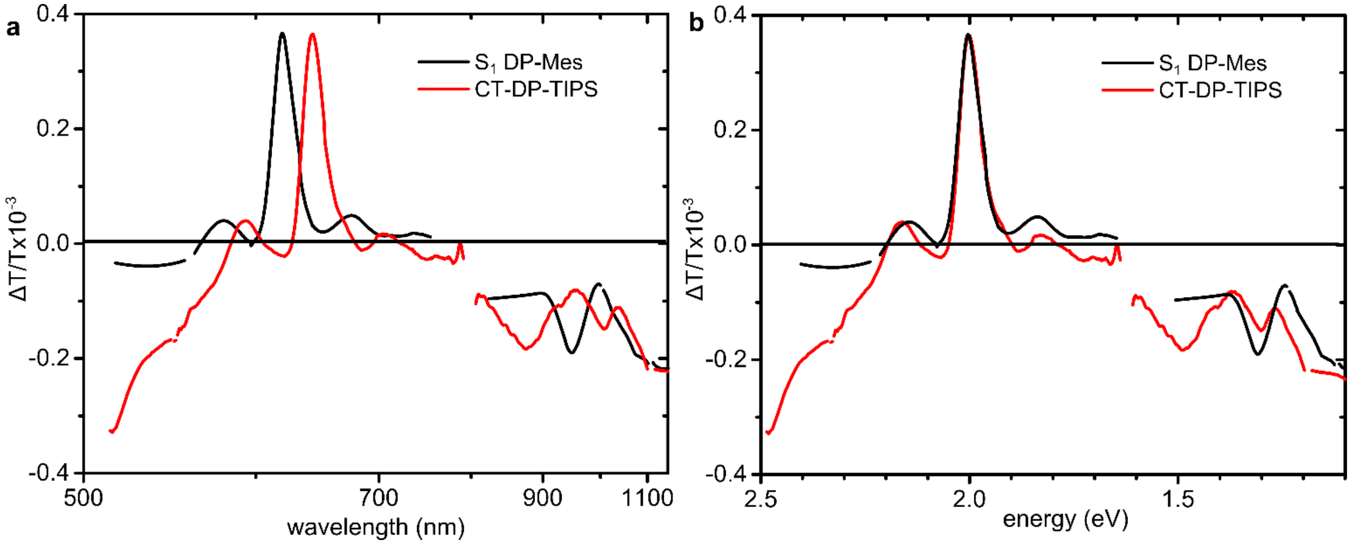

Supplementary Figure 20. S_1-CT mixture *versus* 'pure' CT. (**a**) Comparison of DP-Mes-S_1 state and DP-TIPS- CT excited state spectra, extracted from TA measurements, in (**a**) wavelength scale, and (**b**) energy scale. In the latter the CT spectrum of DP-TIPS has been shifted uniformly so that the GSB peaks align, revealing striking similarity to the DP-Mes singlet PIA spectrum in the NIR. This similarity is presumed to reflect the significant CT component in the initial singlet state in DP-Mes. We note, however, that there are also important differences in the spectral shapes, due to the singlet character of the DP-Mes excited state.

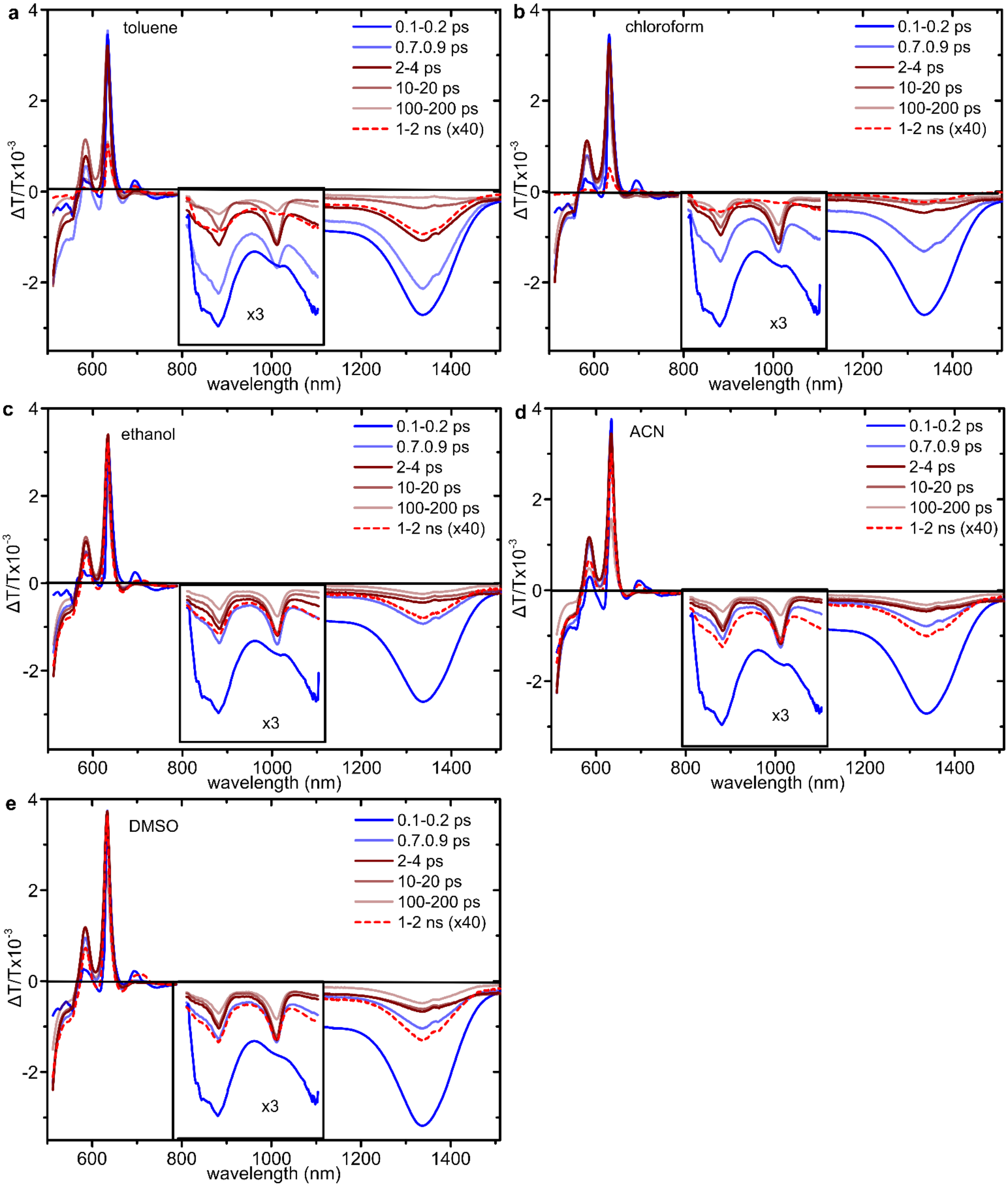

Supplementary Figure 21. Solvent-dependent SF in DP-TIPS. TA spectra of DP-TIPS in (**a**) toluene, (**b**) chloroform, (**c**) ethanol, (**d**) ACN, and (**e**) DMSO at indicated time delays. Dashed line showing spectrum measured on long time-scales and the indicated NIR spectral region have been magnified for clarity.

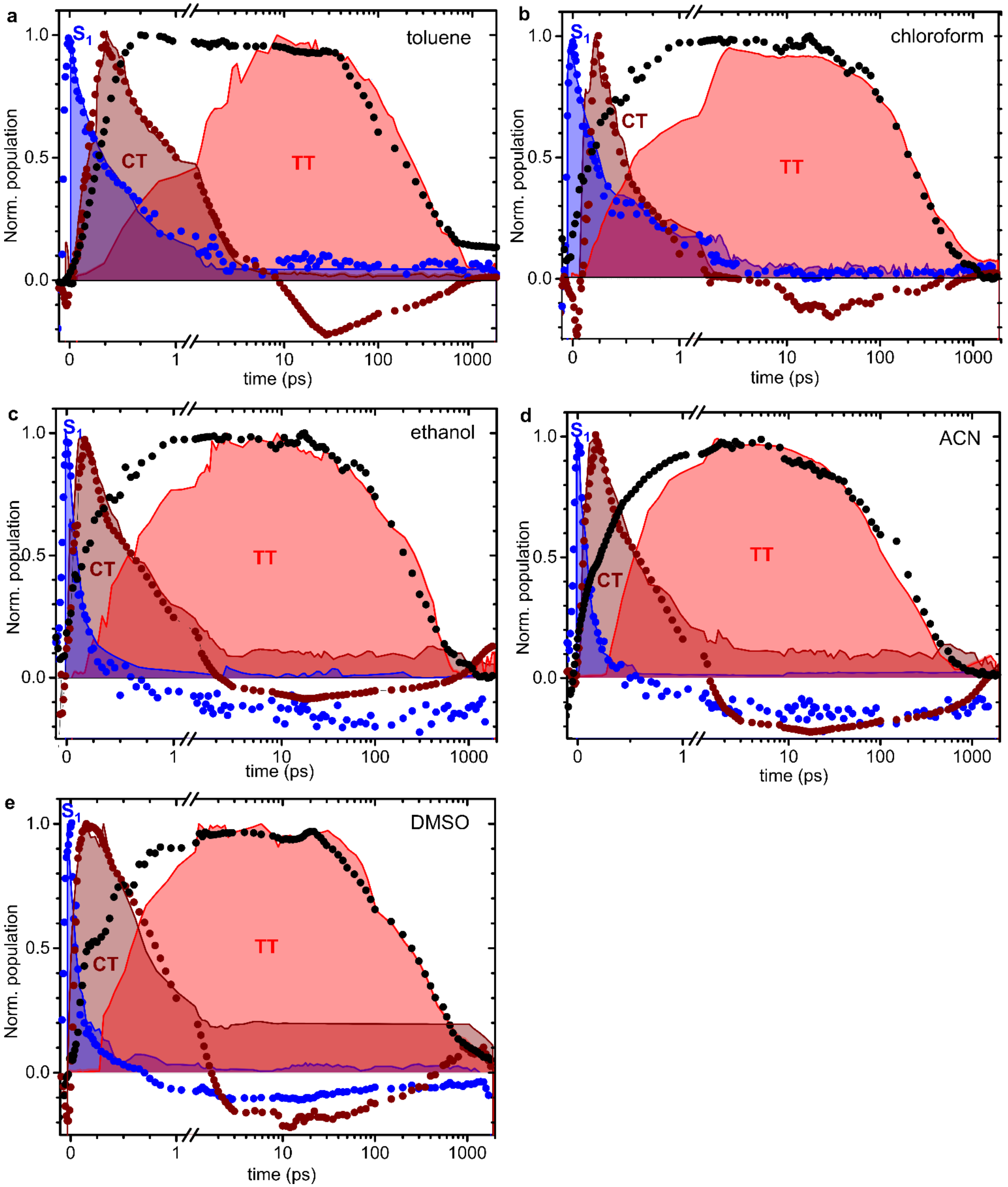

Supplementary Figure 22. Three-species SF dynamics in DP-TIPS. (**a-e**) Normalised population kinetics of different excited species in DP-TIPS in the indicated solvents, extracted from TA spectral decomposition. The scatter plots show TA kinetics of GSB (635-645 nm, black), primarily CT PIA (655-660 nm, wine) and S_1 SE (680-690 nm, blue). The species extracted from spectral decomposition of hexane data are presented in main-text Fig. 5g. Spectral components from other solvent data sets were equivalent.

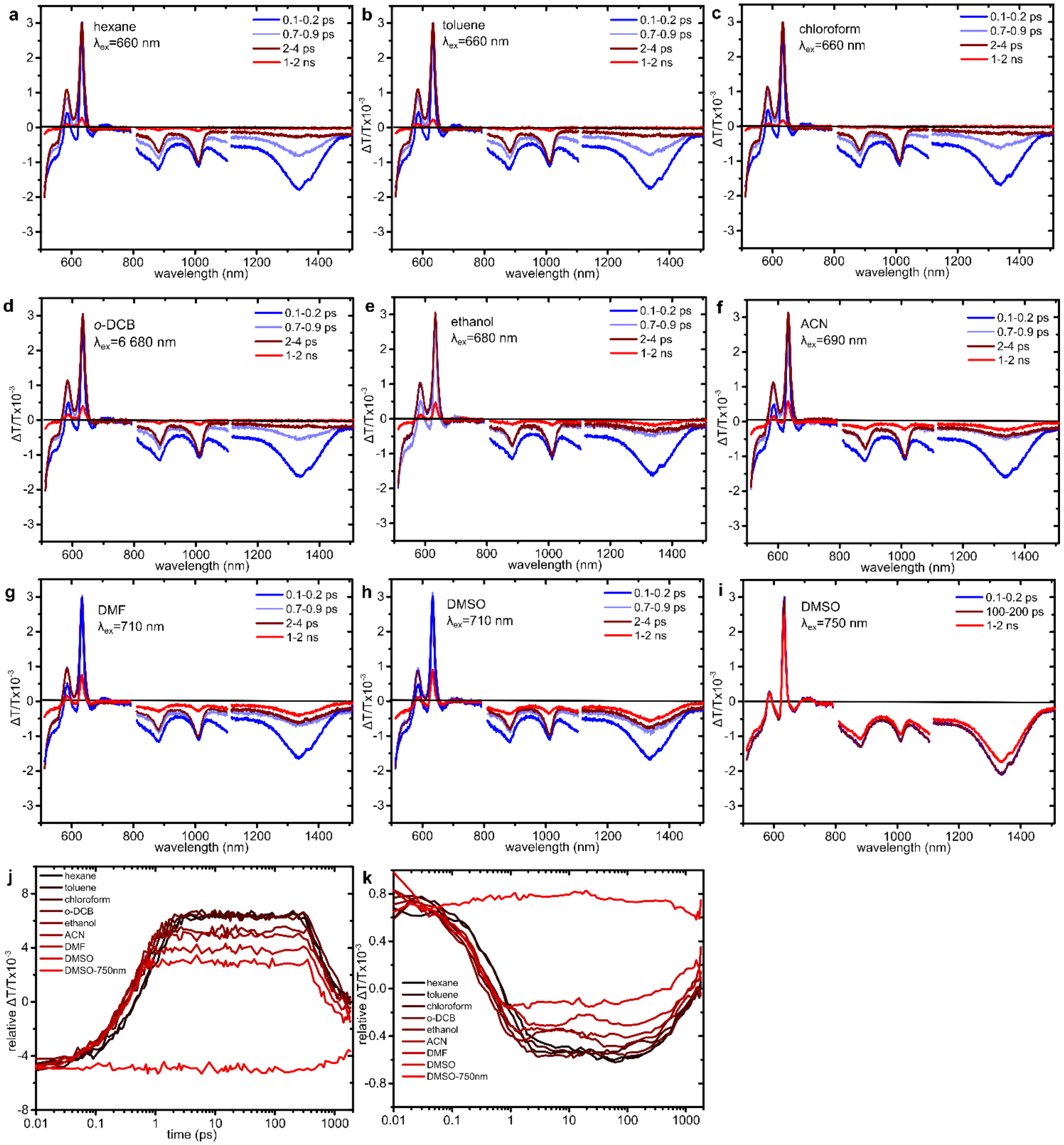

Supplementary Figure 23. Excitation-dependent photophysics of DP-TIPS. TA spectra of DP-TIPS in a range of solvents following sub-bandgap excitation in (**a**) hexane at 660 nm, (**b**) toluene at 660 nm, (**c**) chloroform at 660 nm, (**d**) o-DCB at 680 nm, (**e**) ethanol at 680 nm, (**f**) ACN at 690 nm, (**g**) DMF at 710 nm, (**h**) DMSO at 710 nm and (**i**) DMSO at 750 nm at the indicated time delays. The initial state in all cases can be identified as the CT state from the sharp peaks on either side of the main GSB band and the pronounced PIA peak at 1000 nm. In almost all cases (a-h) the CT state forms at least some population of TT (slightly shifted and narrowed PIA bands in 800-1000 nm and loss of the PIA peak ~1300 nm) on the same timescales observed under resonant excitation. The (**j**) relative kinetics at 615-620 nm (CT PIA) and (**k**) 715-725 nm (CT SE) extracted from the data above show direct CT formation, with decreasing triplet yield (plateau in the kinetics 5-200 ps) as the CT energy level drops with solvent polarity, culminating in no TT formation in DMSO at following 750 nm excitation. All of the kinetics are multiplied by a prefactor to make the GSB at each measurement comparable to the GSB of DP-TIPS in hexane.

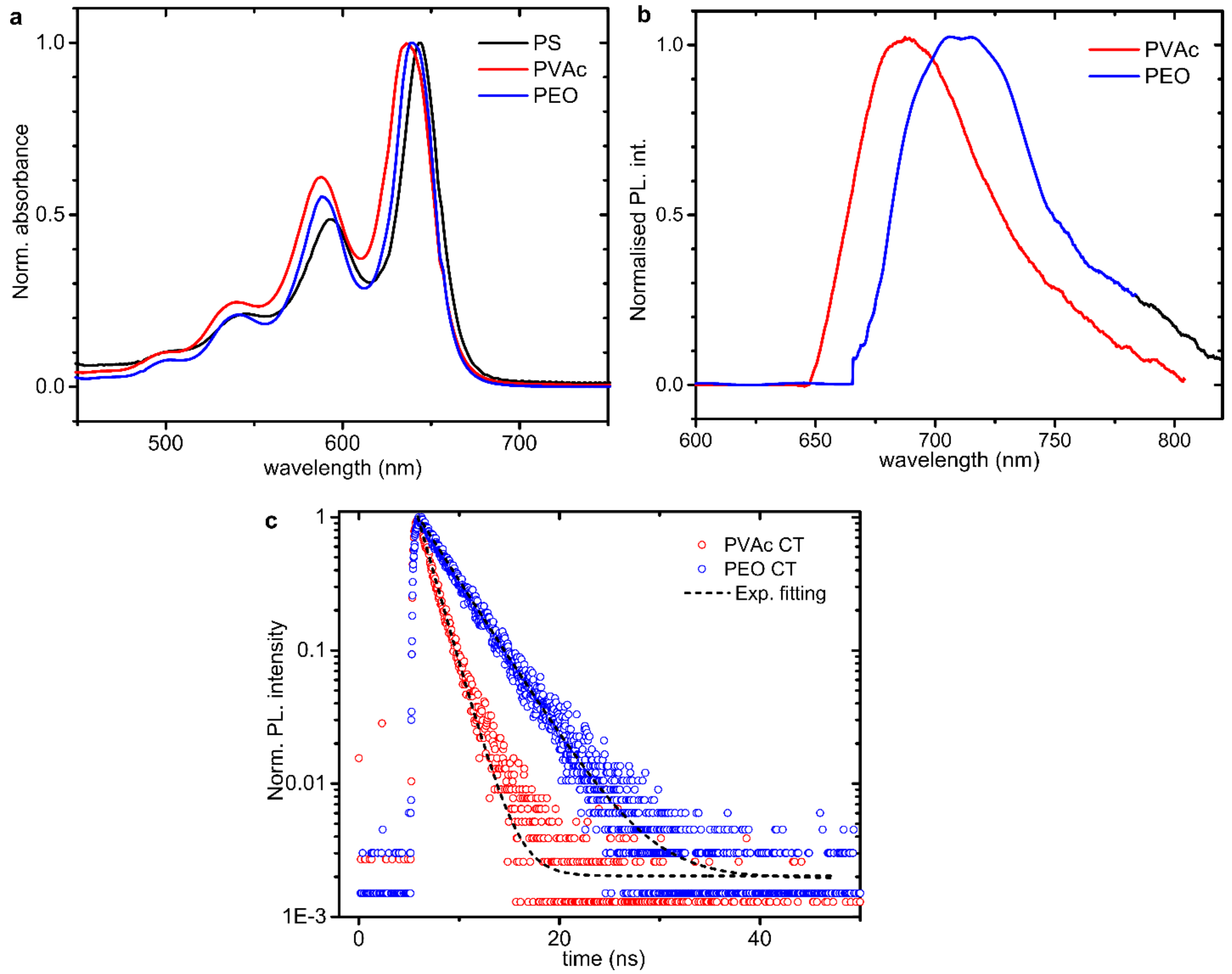

Supplementary Figure 24. Emission from DP-TIPS in polymer matrix. (**a**) Absorption spectra of DP-TIPS embedded in polymer matrices polystyrene (PS), poly-vinylacetate (PVAc) and poly-ethyleneoxide (PEO). (**b**) Photoluminescence spectra of DP-TIPS in PVAc and PEO matrix. Both spectra are dominated by CT emission, whereas the spectrum in PS (main-text Fig. 2e) is completely excitonic, with only a small Stokes shift and well-defined vibronic progression. (**c**) Transient PL kinetic of DP-TIPS in PVAc and PEO matrix. PL decay lifetimes (dashed line) are 1.63 ns in PVAc and 3.68 ns in PEO, in good agreement with the primary CT kinetics determined in TA.

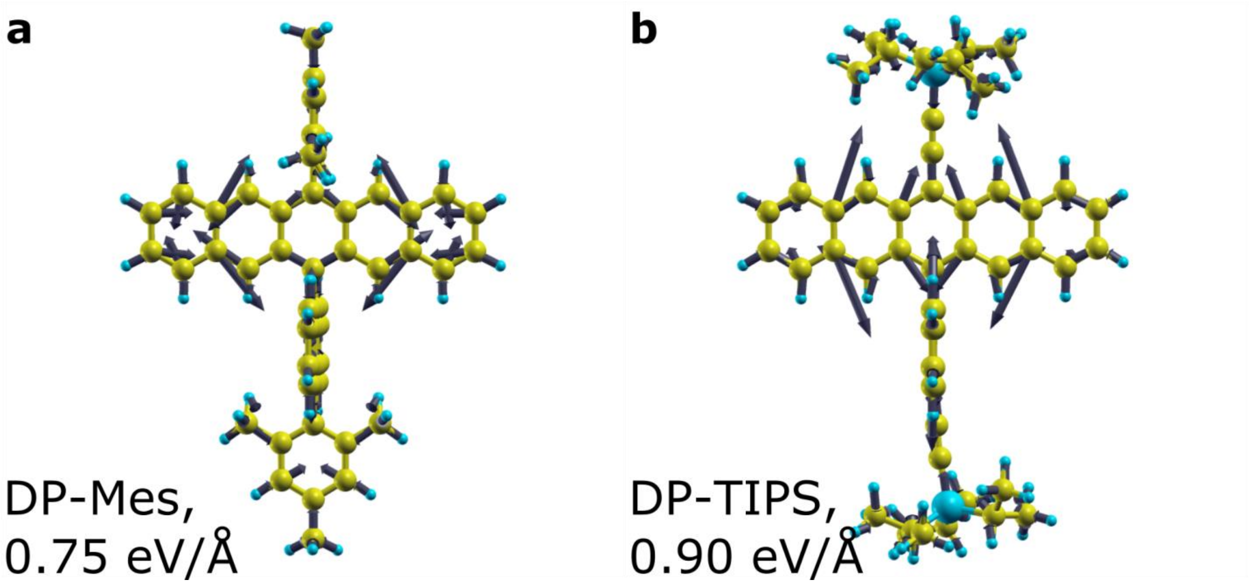

Supplementary Figure 25. S1 relaxation forces in pentacene dimers. Relaxation forces for the vertically excited S1 state were calculated for (a) DP-Mes and (b) DP-TIPS using TDDFT gradients in NWChem. The scale of the forces indicates a tendency towards significant geometric relaxation, particularly in DP-TIPS. The lower overall forces in the -Mes dimer are consistent with the proposed admixture of CT states, which favours the orthogonal geometry.

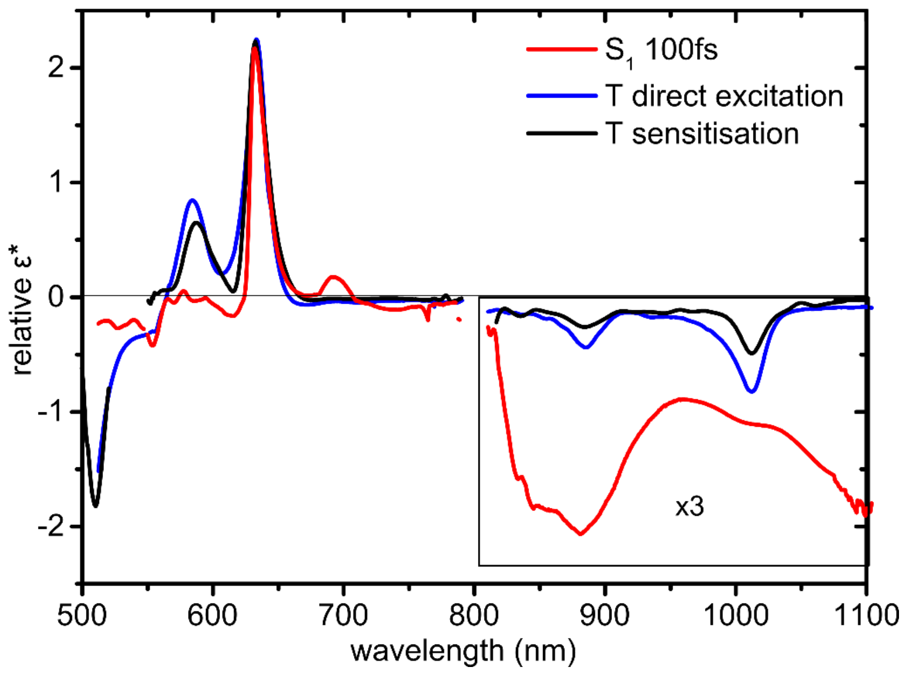

Supplementary Figure 26. Normalisation of DP-TIPS T spectrum (from sensitisation and direct excitation) relative to GSB of S_1 (at 100 fs), used for determination of triplet yields.

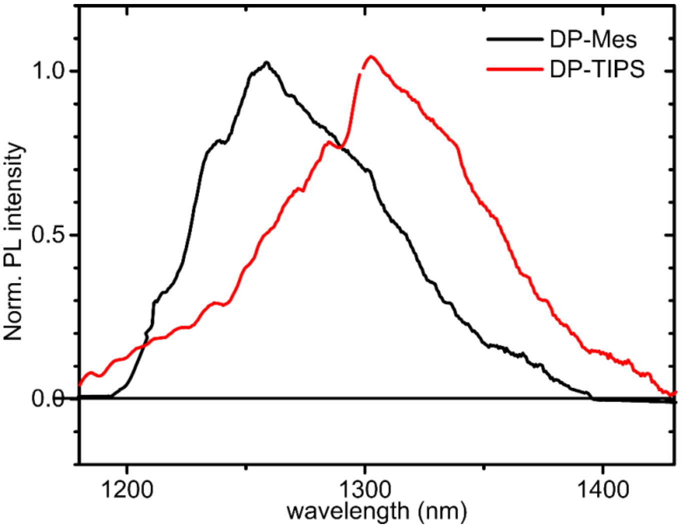

Supplementary Figure 27. Phosphorescence spectra of DP-Mes and DP-TIPS as sensitised by platinum octaethylporphyrin (PtOEP, Sigma Aldrich). Dimer molecules were mixed with PtOEP and dispersed in polystyrene matrix in a weight ratio dimer:PtOEP:polymer of 1:5:95, drop-cast on Spectrosil®. Phosphorescence was detected using a calibrated infrared InGaAs photodiode array (ANDOR iDus 490A) coupled to a spectrograph (ANDOR Shamrock), with CW excitation at 532 nm (0.7 mW).

Supplementary Tables

Supplementary Table 1. Energy levels comparison of LE (TD-DFT) and ET state (c-DFT) of DP-Mes and DP-TIPS in vacuum.

	E_{LE} (TD-DFT) in eV	E_{ET} (c-DFT) in eV	$\|E_{ET-LE}\|$ in eV	Exciton splitting from LE to dark state (eV)
DP-Mes	2.15	2.25	0.10	0.13
DP-TIPS	2.04	2.18	0.14	0.15
P-Mes	2.22	-	-	-
P-TIPS	2.03	-	-	-

Supplementary Table 2. SF rate constant for DP-Mes

Solvent	K_{total} (ps^{-1})	$frac_{SF}$ (from measured triplet yield)	K_{SF} (ps^{-1})	K_{Sstab}(ps^{-1})
Hexane	0.85	0.98	0.84	0.01
Toluene	1.39	0.97	1.34	0.05
Chloroform	2.08	0.89	1.86	0.22
o-DCB	2.56	0.82	2.10	0.47
Ethanol	2.44	0.76	1.85	0.59
Acetonitrile	2.63	0.73	1.92	0.71
DMF	3.44	0.66	2.26	1.18
DMSO	4.02	0.61	2.44	1.56

Supplementary Table 3. SF rate constant for DP-TIPS

Solvent	$K_{S,total}$ (ps^{-1})	$frac_{S\rightarrow CT}$ (from spectral decomposition)	$K_{S\rightarrow CT}$(ps^{-1})	$K_{S,stab}$(ps^{-1})
Hexane	0.88	0.83	0.73	0.15
Toluene	1.19	0.89	1.06	0.13
Chloroform	1.89	0.94	1.77	0.12
o-DCB	2.13	~1	2.13	-
Ethanol	2.33	~1	2.33	-
Acetonitrile	2.44	~1	2.44	-
DMF	2.86	~1	2.86	-
DMSO	3.22	~1	3.22	-

Solvent	$K_{CT,total}$ (ps^{-1})	$frac_{CT\rightarrow TT}$ (from spectral decomposition)	$K_{CT\rightarrow TT}$(ps^{-1})	$K_{CT,stab}$(ps^{-1})
Hexane	1.59	0.93	1.48	0.11
Toluene	1.29	0.95	1.23	0.06

Chloroform	1.64	0.96	1.61	0.03
o- DCB	1.24	0.99	1.23	0.01
Ethanol	2.04	0.91	1.86	0.18
Acetonitrile	1.97	0.88	1.72	0.25
DMF	1.82	0.83	1.50	0.32
DMSO	1.69	0.81	1.38	0.31

Supplementary Table 4. Exponential time constants from global kinetic fit of DP-Mes data in hexane and DMSO solution in main text Fig. 4. τ_3 is determined from measurements using nanosecond excitation and longer pump-probe delays and fixed in the fitting. The time constants are assigned to the decay lifetime of S_1 by SEF (τ_1), the decay of TT by TTA (τ_2), the decay of regenerated S* in hexane SF (τ_3) or radiative decay of residual S_{stab}, which does not undergo SF in DMSO (τ_3).

$\frac{\Delta T}{T} = a_1 * \exp\left(-\frac{t}{\tau_1}\right) + a_2 * \exp\left(-\frac{t}{\tau_2}\right) + a_3 * \exp\left(-\frac{t}{\tau_3}\right)$ τ_1 = 1.31±0.03 ps; τ_2= 757±50 ps; τ_3 = 2.19±0.08 ns			
Hexane	PIA (530-540 nm)	GSB (625-635 nm)	SE (670-680 nm)
a_1	-1.21	0.06	0.92
a_2	1.22	1.06	-0.34
a_3	0.57	0.24	0.19

$\frac{\Delta T}{T} = a_1 * \exp\left(-\frac{t}{\tau_1}\right) + a_2 * \exp\left(-\frac{t}{\tau_2}\right) + a_3 * \exp\left(-\frac{t}{\tau_3}\right)$ τ_1 = 0.25±0.07 ps; τ_2= 546±56 ps; τ_3 = 4.52±0.14 ns			
DMSO	PIA (530-540 nm)	GSB (625-635 nm)	SE (670-680 nm)
a_1	-1.02	0.04	1.03
a_2	0.93	1.05	-0.17
a_3	0.26	0.44	0.34

Supplementary Table 5. Exponential time constants from global kinetic fit of DP-Mes in chloroform, ethanol, ACN, and DMF in Supplementary Figs. 9,10. τ_3 is determined from measurements using nanosecond excitation and longer pump-probe delays and fixed in the fitting. The time constants are assigned to the decay lifetime of S_1 by a combination of SEF and relaxation into S_{stab} (τ_1), the decay of TT via TTA (τ_2), and the radiative decay of the residual S_{stab} (τ_3), which does not undergo SEF.

$\frac{\Delta T}{T} = a_1 * \exp\left(-\frac{t}{\tau_1}\right) + a_2 * \exp\left(-\frac{t}{\tau_2}\right) + a_3 * \exp\left(-\frac{t}{\tau_3}\right)$ τ_1 = 0.46±0.05 ps; τ_2= 649±47 ps; τ_3 = 5.21±0.23 ns			
Chloroform	PIA (530-540 nm)	GSB (625-635 nm)	SE (670-680 nm)
a_1	-1.05	0.03	1.01
a_2	1.03	1.01	-0.31
a_3	0.13	0.13	0.09

$\frac{\Delta T}{T} = a_1 * \exp\left(-\frac{t}{\tau_1}\right) + a_2 * \exp\left(-\frac{t}{\tau_2}\right) + a_3 * \exp\left(-\frac{t}{\tau_3}\right)$ τ_1 = 0.41±0.05 ps; τ_2= 572±37 ps; τ_3 = 4.23±0.16 ns			
Ethanol	PIA (530-540 nm)	GSB (625-635 nm)	SE (670-680 nm)
a_1	-1.11	0.07	1.04
a_2	1.09	1.06	-0.28
a_3	0.17	0.27	0.22

$\frac{\Delta T}{T} = a_1 * \exp\left(-\frac{t}{\tau_1}\right) + a_2 * \exp\left(-\frac{t}{\tau_2}\right) + a_3 * \exp\left(-\frac{t}{\tau_3}\right)$ τ_1 = 0.38±0.06 ps; τ_2= 623±25 ps; τ_3 = 4.23±0.16 ns			
ACN	PIA (530-540 nm)	GSB (625-635 nm)	SE (670-680 nm)
a_1	-1.05	0.11	0.98
a_2	1.06	1.09	-0.21
a_3	0.18	0.31	0.26

$\frac{\Delta T}{T} = a_1 * \exp\left(-\frac{t}{\tau_1}\right) + a_2 * \exp\left(-\frac{t}{\tau_2}\right) + a_3 * \exp\left(-\frac{t}{\tau_3}\right)$ τ_1 = 0.29±0.06 ps; τ_2= 675±43 ps; τ_3 = 5.19±0.13 ns			
DMF	PIA (530-540 nm)	GSB (625-635 nm)	SE (670-680 nm)
a_1	-0.98	0.04	1.04
a_2	1.01	1.02	-0.18
a_3	0.23	0.39	0.31

Supplementary Table 6. Exponential time constants from global kinetic fit of DP-TIPS in solution data in main text Fig. 5 and Supplementary Fig. 11,12. τ_4 is determined from measurements using nanosecond excitation and longer pump-probe delays and fixed in the fitting. The time constants are assigned to the decay lifetime of S_1 by converting into CT and relaxation into S_{stab} (τ_1), the decay of CT (τ_2), the decay of TT (τ_3) and decay of S_{stab} (τ_4).

$\frac{\Delta T}{T} = a_1 * \exp\left(-\frac{t}{\tau_1}\right) + a_2 * \exp\left(-\frac{t}{\tau_2}\right) + a_3 * \exp\left(-\frac{t}{\tau_3}\right) + a_4 * \exp\left(-\frac{t}{\tau_4}\right)$ τ_1 = 1.13±0.02 ps; τ_2 = 1.23±0.03 ps; τ_3 = 847±164 ps; τ_4 = 9.19±0.13 ns			
Hexane	PIA (655-660 nm)	GSB (635-645 nm)	SE (680-690 nm)
a_1	-1.11	0.03	1.03
a_2	1.07	0.06	0.44
a_3	0.13	0.98	-0.22

a_4	0.15	0.26	0.23

$\frac{\Delta T}{T} = a_1 * \exp\left(-\frac{t}{\tau_1}\right) + a_2 * \exp\left(-\frac{t}{\tau_2}\right) + a_3 * \exp\left(-\frac{t}{\tau_3}\right) + a_4 * \exp\left(-\frac{t}{\tau_4}\right)$ τ_1 = 0.84±0.05 ps; τ_2 = 0.77±0.04 ps; τ_3 = 675±58 ps; τ_4 = 10.32±0.31 ns			
Toluene	PIA (655-660 nm)	GSB (635-645 nm)	SE (680-690 nm)
a_1	-1.06	0.02	1.06
a_2	1.02	0.05	0.35
a_3	0.14	1.03	-0.26
a_4	0.13	0.22	0.17

$\frac{\Delta T}{T} = a_1 * \exp\left(-\frac{t}{\tau_1}\right) + a_2 * \exp\left(-\frac{t}{\tau_2}\right) + a_3 * \exp\left(-\frac{t}{\tau_3}\right) + a_4 * \exp\left(-\frac{t}{\tau_4}\right)$ τ_1 = 0.53±0.06 ps; τ_2 = 0.61±0.04 ps; τ_3 = 524±81 ps; τ_4 = 11.09±0.22 ns			
Chloroform	PIA (655-660 nm)	GSB (635-645 nm)	SE (680-690 nm)
a_1	-1.07	0.06	1.05
a_2	1.02	0.03	0.29
a_3	0.15	1.03	-0.31
a_4	0.02	0.14	0.08

Supplementary Table 7. Exponential time constants from global kinetic fit of DP-TIPS in solution data in main text Fig. 5 and Supplementary Fig. 11,12. τ_4 is determined from measurements using nanosecond excitation and longer pump-probe delays and is fixed in the fitting. The time constants are assigned to the decay lifetime of S_1 by converting into CT (τ_1), the decay of CT (τ_2), the decay of TT (τ_3) and decay of non-SF CT (τ_4).

$\frac{\Delta T}{T} = a_1 * \exp\left(-\frac{t}{\tau_1}\right) + a_2 * \exp\left(-\frac{t}{\tau_2}\right) + a_3 * \exp\left(-\frac{t}{\tau_3}\right) + a_4 * \exp\left(-\frac{t}{\tau_4}\right)$ τ_1 = 0.47±0.05 ps; τ_2= 0.85±0.04 ps; τ_3 = 633±47 ps; τ_4 = 4.78±0.18 ns			
o-DCB	PIA (655-660 nm)	GSB (635-645 nm)	SE (680-690 nm)
a_1	-1.04	0.04	1.02
a_2	1.05	0.06	0.27
a_3	0.20	1.08	-0.26
a_4	0.04	0.03	0.04

$\frac{\Delta T}{T} = a_1 * \exp\left(-\frac{t}{\tau_1}\right) + a_2 * \exp\left(-\frac{t}{\tau_2}\right) + a_3 * \exp\left(-\frac{t}{\tau_3}\right) + a_4 * \exp\left(-\frac{t}{\tau_4}\right)$ τ_1 = 0.43±0.07 ps; τ_2= 0.49±0.06 ps; τ_3 = 539±72 ps; τ_4 = 5.12±0.20 ns

ethanol	PIA (655-660 nm)	GSB (635-645 nm)	SE (680-690 nm)
a_1	-1.02	0.06	0.95
a_2	0.95	0.03	0.21
a_3	0.23	0.97	-0.21
a_4	0.07	0.09	0.06

$\frac{\Delta T}{T} = a_1 * \exp\left(-\frac{t}{\tau_1}\right) + a_2 * \exp\left(-\frac{t}{\tau_2}\right) + a_3 * \exp\left(-\frac{t}{\tau_3}\right) + a_4 * \exp\left(-\frac{t}{\tau_4}\right)$ τ_1 = 0.41±0.04 ps; τ_2= 0.51±0.05 ps; τ_3 = 609±49 ps; τ_4 = 4.23±0.27 ns			
ACN	PIA (655-660 nm)	GSB (635-645 nm)	SE (680-690 nm)
a_1	-0.97	0.02	1.05
a_2	1.03	0.07	0.24
a_3	0.24	1.04	-0.17
a_4	0.09	0.12	0.09

$\frac{\Delta T}{T} = a_1 * \exp\left(-\frac{t}{\tau_1}\right) + a_2 * \exp\left(-\frac{t}{\tau_2}\right) + a_3 * \exp\left(-\frac{t}{\tau_3}\right) + a_4 * \exp\left(-\frac{t}{\tau_4}\right)$ τ_1 = 0.35±0.08 ps; τ_2= 0.55±0.04 ps; τ_3 = 678±84 ps; τ_4 = 3.98±0.49 ns			
DMF	PIA (655-660 nm)	GSB (635-645 nm)	SE (680-690 nm)
a_1	-1.01	0.03	0.99
a_2	0.96	0.06	0.33
a_3	0.27	1.09	-0.12
a_4	0.13	0.17	0.11

$\frac{\Delta T}{T} = a_1 * \exp\left(-\frac{t}{\tau_1}\right) + a_2 * \exp\left(-\frac{t}{\tau_2}\right) + a_3 * \exp\left(-\frac{t}{\tau_3}\right) + a_4 * \exp\left(-\frac{t}{\tau_4}\right)$ τ_1 = 0.31±0.06 ps; τ_2= 0.61±0.05 ps; τ_3 = 691±55 ps; τ_4 = 4.76±0.38 ns			
DMSO	PIA (655-660 nm)	GSB (635-645 nm)	SE (680-690 nm)
a_1	-0.92	0.05	1.12
a_2	1.07	0.07	0.42
a_3	0.31	1.05	-0.09
a_4	0.17	0.20	0.14

Supplementary Table 8. Exponential time constants from global kinetic fit of DP-TIPS in PS matrix data in main text Fig. 7.

The time constants are assigned to the decay lifetime of S_1 by SF (τ_1), and the decay of TT (τ_2).

$\frac{\Delta T}{T} = a_1 * \exp\left(-\frac{t}{\tau_1}\right) + a_2 * \exp\left(-\frac{t}{\tau_2}\right)$ τ_1 = 0.94±0.03 ps; τ_2= 1.61±0.12 ns			
PS	PIA (655-660 nm)	GSB (635-645 nm)	SE (680-690 nm)
a_1	-0.02	0.03	1.04

a_2	0.03	1.06	-0.22

Supplementary Table 9. Exponential time constants from global kinetic fit of DP-TIPS in PVAc and PEO matrix data in main text Fig. 7. τ_4 is determined from measurements using nanosecond excitation and longer pump-probe delays and is fixed in the fitting. The time constants are assigned to the decay lifetime of S_1 by converting into CT (τ_1), the decay of CT (τ_2), the decay of TT (τ_3) and decay of non-SF CT (τ_4).

$\frac{\Delta T}{T} = a_1 * \exp\left(-\frac{t}{\tau_1}\right) + a_2 * \exp\left(-\frac{t}{\tau_2}\right) + a_3 * \exp\left(-\frac{t}{\tau_3}\right) + a_4 * \exp\left(-\frac{t}{\tau_4}\right)$ τ_1 = 0.23±0.05 ps; τ_2= 1.66±0.14 ps; τ_3 = 1.81±0.08 ns; τ_4 = 1.81±0.21 ns			
PVAc	PIA (655-660 nm)	GSB (635-645 nm)	SE (680-690 nm)
a_1	-0.97	0.06	0.76
a_2	0.99	0.01	0.39
a_3	0.14	0.97	-0.17
a_4	0.35	0.33	0.24

$\frac{\Delta T}{T} = a_1 * \exp\left(-\frac{t}{\tau_1}\right) + a_2 * \exp\left(-\frac{t}{\tau_2}\right) + a_3 * \exp\left(-\frac{t}{\tau_3}\right) + a_4 * \exp\left(-\frac{t}{\tau_4}\right)$ τ_1 = 0.12±0.07 ps; τ_2= 2.37±0.32 ps; τ_3 = 0.24±0.03 µs; τ_4 = 3.34±0.17ns			
PEO	PIA (655-660 nm)	GSB (635-645 nm)	SE (680-690 nm)
a_1	-1.04	0.07	0.32
a_2	1.01	0.02	0.41
a_3	0.08	1.04	-0.06
a_4	0.77	0.73	0.24

Supplementary Notes

Supplementary Note 1: Electronic structure calculations

In order to perform ground and excited state calculations, we employ linear-scaling Density Functional Theory as implemented in the ONETEP code.[1] Linear-scaling DFT is based on the single-electron density matrix (rather than Kohn-Sham orbitals). The density matrix is expanded in a basis of localised, atom-centred functions called NGWFs (non-orthogonal generalised Wannier functions).[2] The matrix of expansion coefficients is called the density kernel. The support of the NGWFs is strictly confined to a chosen localisation radius (a convergence parameter).

In ONETEP, a conjugate gradients optimisation scheme is used to minimise the total energy with respect to both the density kernel (subject to the constraints of idempotency and normalisation), and the NGWFs. The NGWFs are optimised in an underlying basis set of "psinc" functions, i.e. delta functions commensurate with the simulation box and with a finite cutoff. These have the desirable property of being equivalent to plane waves.

The central idea of constrained-DFT (c-DFT) is to add terms to the DFT total energy functional that impose desired constraints on the charge density of a system.[3,4] In the case of intramolecular CT states of pentacene dimers, these constraints are one fewer charge on the donor unit and one additional charge on the acceptor unit, relative to the ground state.

In this work we impose the constraints using monomer-localised projection operators to partition the density. Within the linear-scaling DFT framework it is a natural choice to make use of the localised NGWFs to define projection operators.[5] Here, we resort to a fixed set of NGWFs from a ground-state calculation for this purpose.

To obtain CT excitation energies, we first optimise the molecular geometry in the ground state (resulting in an orthogonal configuration with approximate D2d symmetry for both pentacene dimers). This gives both a total energy for the relaxed ground state and a set of converged ground-state NGWFs which are then employed as cDFT projectors. Vertical CT excitation energies are calculated as the difference between the constrained total energy and the ground state total energy.

To incorporate the electrostatic effects of different solvent environments, we employ an implicit solvent model with open boundary conditions.[6] In the ONETEP implementation the molecule occupies a smooth dielectric cavity in an otherwise uniform, infinite dielectric. The nonhomogeneous Poisson equation is solved to obtain the potential due to the molecular density in the dielectric.

For all DFT calculations we use the LDA functional and norm-conserving pseudopotentials. The energy cutoff is set to 750 eV which results in well-converged energies for organic molecules using the pseudopotentials we employ here. We use 1 NGWF per hydrogen atom and 4 NGWFs per carbon/silicon atom. For the truncation radius of the NGWFs a value of 10 Bohr is chosen. At the start of the calculations the NGWFs are initialised to pseudoatomic orbitals and then optimised in terms of the psinc basis.[7]

Furthermore we performed Time Dependent-DFT (TD-DFT) calculations of the locally excited (LE) states with the NWChem code at the cc-PVDZ/LC-BLYP level of theory. This yielded vacuum energies of 2.15 eV and 2.04 eV for DP-Mes and DP-TIPS, respectively. In comparison with the cDFT results this indicates that the CT-LE gap in DP-Mes is distinctly smaller than in DP-TIPS (0.10 eV vs 0.14 eV in vacuum).

Supplementary Note 2: Triplet yield calculation

As described in the Methods section, the triplet yield was determined from the ratio of S_1 to T_1 molar extinction coefficient, which was in turn established through normalization against the GSB region 635-640 nm. The calculations for DP-Mes and DP-TIPS take the extinction ratio from previous publication[1] and Fig. S19 respectively. The peak S_1 population was taken to be at the 100 fs time-slice, while the T_1 value was taken at 10 ps (any delay in the range ~5 ps to ~50 ps gives equivalent results).

$$\Phi_{\text{Triplet}} = \frac{\Delta A(T_1)|_{t=10\ ps}}{\Delta A(S_1)|_{t=100\ fs}} \times \frac{\varepsilon^*_{S1}}{\varepsilon^*_{T1}}$$

For DP-Mes in hexane, calculation at ~ 525 nm:

$$\Phi_{\text{Triplet}} = \frac{(3.69 \times 10^{-3})(1.2\ Lmol^{-1}cm^{-1})}{(2.26 \times 10^{-3})(1.0\ Lmol^{-1}cm^{-1})} \approx 1.96$$

Calculation at ~ 910 nm:

$$\Phi_{\mathrm{Triplet}} = \frac{(8.73 \times 10^{-4})(1.4\ Lmol^{-1}cm^{-1})}{(6.28 \times 10^{-4})(1.0\ Lmol^{-1}cm^{-1})} \approx 1.95$$

Calculation at ~940 nm:

$$\Phi_{\mathrm{Triplet}} = \frac{(6.11 \times 10^{-4})(3.2\ Lmol^{-1}cm^{-1})}{(9.78 \times 10^{-4})(1.0\ Lmol^{-1}cm^{-1})} \approx 1.94$$

Calculation at ~1380 nm:

$$\Phi_{\mathrm{Triplet}} = \frac{(1.63 \times 10^{-3})(2.1\ Lmol^{-1}cm^{-1})}{(1.73 \times 10^{-3})(1.0\ Lmol^{-1}cm^{-1})} \approx 1.98$$

Reported value, 1.97, is the average of the four values calculated above.

For DP-Mes in DMSO, calculation at ~ 525 nm:

$$\Phi_{\mathrm{Triplet}} = \frac{(2.21 \times 10^{-3})(1.2\ Lmol^{-1}cm^{-1})}{(2.12 \times 10^{-3})(1.0\ Lmol^{-1}cm^{-1})} \approx 1.25$$

Calculation at ~ 910 nm:

$$\Phi_{\mathrm{Triplet}} = \frac{(4.81 \times 10^{-4})(1.4\ Lmol^{-1}cm^{-1})}{(6.01 \times 10^{-4})(1.0\ Lmol^{-1}cm^{-1})} \approx 1.14$$

Calculation at ~940 nm:

$$\Phi_{\mathrm{Triplet}} = \frac{(3.67 \times 10^{-4})(3.2\ Lmol^{-1}cm^{-1})}{(9.17 \times 10^{-4})(1\ Lmol^{-1}cm^{-1})} \approx 1.27$$

Calculation at ~1380 nm:

$$\Phi_{\mathrm{Triplet}} = \frac{(9.08 \times 10^{-4})(2.1\ Lmol^{-1}cm^{-1})}{(1.62 \times 10^{-3})(1.0\ Lmol^{-1}cm^{-1})} \approx 1.18$$

For triplet yield calculated in solvent with S_{stab}, the $\Delta A(T_1)$ at 10 ps used in the calculation is corrected for the underlying $\Delta A(S_{stab})$ that does not undergo singlet fission. The yield is still calculated against the full initial singlet population.

For DP-TIPS in hexane, calculation at ~ 520 nm:

$$\Phi_{\mathrm{Triplet}} = \frac{(5.43 \times 10^{-4})(0.4\ Lmol^{-1}cm^{-1})}{(1.28 \times 10^{-4})(1.0\ Lmol^{-1}cm^{-1})} \approx 1.66$$

Calculation at ~880 nm:

$$\Phi_{\mathrm{Triplet}} = \frac{(2.68 \times 10^{-4})(8.2\ Lmol^{-1}cm^{-1})}{(1.24 \times 10^{-3})(1.0\ Lmol^{-1}cm^{-1})} \approx 1.77$$

Calculation at ~1015 nm:

$$\Phi_{\text{Triplet}} = \frac{(7.17 \times 10^{-4})(2.3\ Lmol^{-1}cm^{-1})}{(9.10 \times 10^{-4})(1.0\ Lmol^{-1}cm^{-1})} \approx 1.81$$

Reported value, 1.77, is the average of the four values calculated above.

For DP-TIPS in *o*-DCB, calculation at ~ 520 nm:

$$\Phi_{\text{Triplet}} = \frac{(6.19 \times 10^{-4})(0.4\ Lmol^{-1}cm^{-1})}{(1.28 \times 10^{-4})(1.0\ Lmol^{-1}cm^{-1})} \approx 1.87$$

Calculation at ~880 nm:

$$\Phi_{\text{Triplet}} = \frac{(2.93 \times 10^{-4})(8.2\ Lmol^{-1}cm^{-1})}{(1.25 \times 10^{-3})(1.0\ Lmol^{-1}cm^{-1})} \approx 1.94$$

Calculation at ~1015 nm:

$$\Phi_{\text{Triplet}} = \frac{(6.96 \times 10^{-4})(2.3\ Lmol^{-1}cm^{-1})}{(8.30 \times 10^{-4})(1.0\ Lmol^{-1}cm^{-1})} \approx 1.92$$

For DP-TIPS in DMF, calculation at ~ 520 nm:

$$\Phi_{\text{Triplet}} = \frac{(1.47 \times 10^{-3})(0.4\ Lmol^{-1}cm^{-1})}{(3.38 \times 10^{-4})(1.0\ Lmol^{-1}cm^{-1})} \approx 1.74$$

Calculation at ~880 nm:

$$\Phi_{\text{Triplet}} = \frac{(2.51 \times 10^{-4})(8.2\ Lmol^{-1}cm^{-1})}{(1.26 \times 10^{-3})(1.0\ Lmol^{-1}cm^{-1})} \approx 1.63$$

Calculation at ~1015 nm:

$$\Phi_{\text{Triplet}} = \frac{(5.32 \times 10^{-4})(2.3\ Lmol^{-1}cm^{-1})}{(7.88 \times 10^{-4})(1.0\ Lmol^{-1}cm^{-1})} \approx 1.55$$

For triplet yield calculated in solvent with residual S_{stab} or CT, the $\Delta A(T_1)$ at 10 ps used in the calculation is corrected for the underlying $\Delta A(S_{stab})$ or $\Delta A(CT)$ that does not undergo singlet fission. The yield is still calculated against the full initial singlet population.

Supplementary References

1 Skylaris, C.-K., Haynes, P. D., Mostofi, A. A. & Payne, M. C. Introducing ONETEP: Linear-scaling density functional simulations on parallel computers. *J. Chem Phys.* **122**, 084119 (2005).

2 Skylaris, C.-K., Mostofi, A. A., Haynes, P. D., Diéguez, O. & Payne, M. C. Nonorthogonal generalized Wannier function pseudopotential plane-wave method. *Phys.Rev. B* **66**, 035119 (2002).

3 Dederichs, P. H., Blügel, S., Zeller, R. & Akai, H. Ground states of constrained systems: Application to cerium impurities. *Phys. Rev. Lett.* **53**, 2512-2515 (1984).

4 Kaduk, B., Kowalczyk, T. & Van Voorhis, T. Constrained density functional theory. *Chem. Revs* **112**, 321-370 (2012).

5 O'Regan, D. D., Hine, N. D. M., Payne, M. C. & Mostofi, A. A. Projector self-consistent DFT+*U* using nonorthogonal generalized Wannier functions. *Phys. Rev. B* **82**, 081102 (2010).

6 Dziedzic, J., Helal, H. H., Skylaris, C. K., Mostofi, A. A. & Payne, M. C. Minimal parameter implicit solvent model for ab initio electronic-structure calculations. *EPL (Europhysics Letters)* **95**, 43001 (2011).

7 Ruiz-Serrano, Á., Hine, N. D. & Skylaris, C.-K. Pulay forces from localized orbitals optimized in situ using a psinc basis set. *J. Chem. Phys.* **136**, 234101 (2012).
